# Electrochemical CO_2_ Capture, Release, and Reduction by a Benzothiadiazole Molecule with Multiple Redox States

**DOI:** 10.1002/cssc.202501724

**Published:** 2025-10-16

**Authors:** Martin Axelsson, Carlos Enrique Torres‐Mendez, Mun Hon Cheah, Haining Tian

**Affiliations:** ^1^ Physical Chemistry Department of Chemistry‐Ångström Laboratory Uppsala University Box 523 75120 Uppsala Sweden; ^2^ Molecular Biomimetics Department of Chemistry‐Ångström Laboratory Uppsala University Box 523 75120 Uppsala Sweden

**Keywords:** benzothiadiazole, CO_2_ captures, CO_2_ releases, electrochemistry, infrared spectroscopy

## Abstract

Using small organic molecular redox carriers to reversibly capture CO_2_ and convert it to carbon‐based chemicals is a promising approach to mitigate the ongoing climate crisis. 2,1,3‐benzothiadiazole (BT) is an interesting unit due to its proven interaction with CO_2_ upon reduction and the ease of tuning its structure. In this work, by introducing two CN in BT, the molecule 2,1,3‐benzothiadiazole‐4,7‐dicarbonitrile (BTDN) has multiple reduced states as compared to BT and is found to interact with CO_2_ at multiple reduced states. The work is carried out with a combination of (spectro‐)electrochemical and computational studies. Cyclic voltammetry experiments in the presence of CO_2_ show a clear interaction between BTDN and CO_2_ upon the second reduction of BTDN and a large current increase at the third reduction. Density functional theory calculations prove a large variety of possible CO_2_‐bound species that can match the experimental data. The binding of CO_2_ on BTDN is found to be reversible upon the oxidation of the species, especially with low concentrations of CO_2_. From NMR and IR experiments, certain amount of reduced product – oxalate is detected after bulk electrolysis at the third reduction potential in the presence of CO_2_, showing the potential toward electrocatalysis after structural tuning and systematical optimization.

## Graphical abstract

Upon reduction, the molecule 2,1,3‐benzothiadiazole‐4,7‐dicarbonitrile (BTDN) can react and capture CO_2_ forming a variety of adducts. Electrochemistry, spectroelectrochemistry, and density functional theory calculations are employed to shine light into the nature of these adducts. Under highly reductive conditions oxalate formation is observed in the presence of BTDN and CO_2_ in acetonitrile.

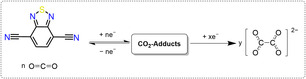

## 1 Introduction

With the growing climate impact of increasing concentration of CO_2_ in the atmosphere, it is clear that the emission of CO_2_ needs to be brought to a complete stop. This will be an especially challenging transition since many of the processes that emit CO_2_ will still need to continue for a functioning society. To be able to achieve these crucial steps, technologies need to be developed to both capture CO_2_ and convert it into useful products such as fuels and feedstocks.^[^ ^1^ ^]^ Considering the scale at which these technologies need to operate on they need to be made of cheap and accessible materials that can be available all over the world. One of the approaches that could fit the requirements is the use of molecular redox carriers to bind up and release the CO_2_ or even form new chemicals at different redox states.^[^ ^2^, ^3^, ^4^ ^]^ This approach is appealing due to the mild environments of electrochemical methods compared to commonly used thermochemical alternatives, as well as the ease of tuning the properties of this class of molecules.^[^ ^5^ ^]^ Ideally, the synthetic routes to these molecular redox carriers should be simple and they should be prepared from cheap and abundant elements. A group of molecules that fits these requirements is that of small organic redox‐active molecules.^[^ ^3^, ^6^ ^]^ Typical binding modes to CO_2_ can be through free electron pairs of either O or N or to the *π−π* interaction of conjugated bonds, as shown in several published systems, with examples such as quinones,^[^ ^7^, ^8^, ^9^, ^10^, ^11^, ^12^ ^]^ pyridines,^[^ ^13^, ^14^, ^15^ ^]^ and thiols.^[^ ^16^, ^17^ ^]^ Moreover, very similar types of molecules with highly conjugated nitrogen‐rich systems, for example, benzimidazoles have been used as hydride donor that facilitate electrochemical reduction of CO_2_ to formic acid^[^ ^2^, ^18^, ^19^ ^]^ and when used in cascade catalytic systems can produce methanol under photoelectrochemical conditions.^[^ ^20^ ^]^ A triazole compound has shown electrocatalytic activity for CO_2_ reduction into CH_4_ and CO.^[^ ^21^ ^]^ Combining both CO_2_ capture and reduction in a single‐molecular system is an attractive approach. In this case, a molecule can be used to capture CO_2_ in one redox state and function as an electrocatalyst for reduction at a second redox state.

**Scheme 1:**
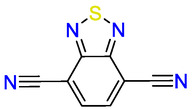
The chemical structure of 2,1,3‐benzothiadiazole 4,7‐dicarbonitrile.

Recently, a compound called 2,1,3‐benzothiadiazole (BT) has been shown to bind two CO_2_ molecules upon reduction at ≈−1.9 V versus Fc^0^^/+^ in a two‐electron process.^[^ ^22^, ^23^ ^]^ In previous work from our group, a similar molecule called 2,1,3‐benzothiadiazole‐4,7‐dicarbonitrile (BTDN, **Scheme** **1**) was shown to bind with protons and upon subsequent reduction, carry on catalytic hydrogen evolution.^[^ ^24^ ^]^ Due to the two strongly electron‐withdrawing nitrile groups, it is much easier to reduce BTDN in comparison to the mother compound BT. The first reduction of BTDN is achieved at −1.06 V versus Fc^0/+^ and the second reduction at −1.89 V versus Fc^0/+^. For comparison, the first reduction of BT is found at −1.87 V versus Fc^0/+^.^[^ ^23^ ^]^ This encouraged us to further study the interaction between BTDN at its different reduced states and CO_2_ as well as to explore the potential catalytic ability for CO_2_ reduction in this work.

## 2 Results and Discussion

### 2.1 Spectroelectrochemical Study of BTDN Reduction in Presence of Ar

**Figure 1:**
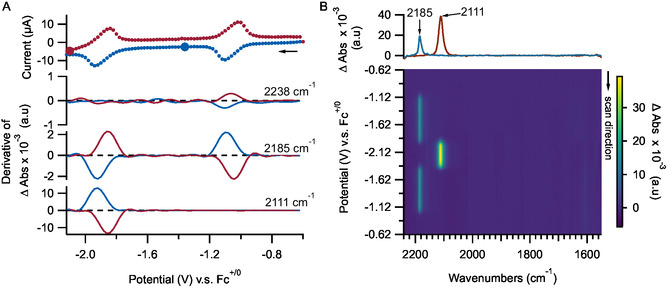
IR‐SEC of 2 mM solution of BTDN in acetonitrile with 0.2 M TBAPF_6_ supporting electrolyte under 3 bar Ar. The potential step is 20 mV and the average scan rate is 15 mV s^−1^. A) Top panel: current–voltage response during IR‐SEC experiment. Bottom panels: derivative of peak intensity of ν(CN) bands as a function of applied potential. Blue traces indicate cathodic scans, red traces indicate anodic scans. B) Top panel: representative IR spectra recorded during IR‐SEC where the applied potential is color coded based on color of each spectrum matching the large circular symbol in panel A. Bottom panel: 2D‐image plot of IR spectra as a function of applied potential.

Under Ar atmosphere, the current response from scanning spectroelectrochemistry (SEC) is fully reversible with two reversible reductions at −1.06 and −1.89 V versus Fc^0/+^ in MeCN,^[^ ^24^ ^]^ consistent with previously reported cyclic voltammetry (CV) experiments. The species formed by the reduction of BTDN in a one‐electron fashion generated a new peak at 2185 cm^−1^ and a bleach at 2238 cm^−1^ in the IR spectra (**Figure** **1**). This is assigned to the shift of the C≡N stretching mode as electron density is accumulated on the molecule, it also fits the mode that we have previously assigned to the BTDN^•−^ species.^[^ ^24^ ^]^ In the second reduction wave the peak shifts to even lower wavenumbers, the peak appears at 2111 cm^−1^ and is assigned to the BTDN^2−^ dianion.

### 2.2 Spectroelectrochemical Study of BTDN Reduction in Presence of CO_2_

**Figure 2:**
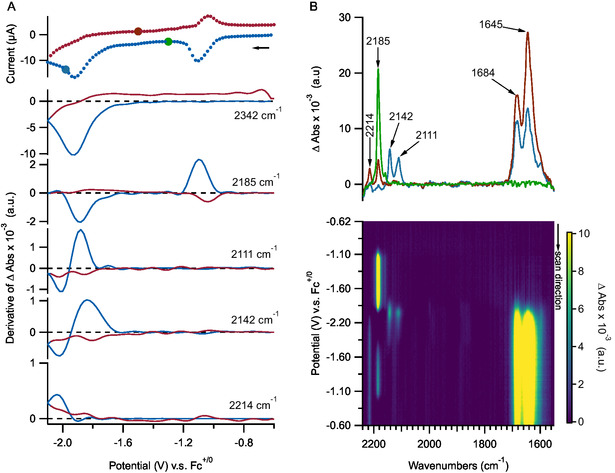
IR‐SEC of 2 mM solution of BTDN in acetonitrile with 0.2 M TBAPF_6_ supporting electrolyte under 2 bar 0.8% CO_2_ mixture with Ar. The potential step is 20 mV and the average scan rate is 12 mV s^−1^. A) Top panel: current–voltage response during IR‐SEC experiment. Bottom panels: derivative of peak intensity of ν(CN) bands as a function of applied potential. Blue traces indicate cathodic scans, red traces indicate anodic scans. B) Top panel: representative IR spectra recorded during IR‐SEC where the applied potential is color coded based on color of each spectrum matching the large circular symbol in panel A. Bottom panel: 2D image plot of IR spectra as function of applied potential. The false‐color image on the bottom right panel is capped at Δ abs value of 10 × 10^−3^ to aid visualization of bands with weaker intensity.

In the presence of 2 bar of 0.8% CO_2_ (corresponding to 4.32 mM CO_2_), reduction of BTDN to BTDN^•−^ appears unaffected by the presence of CO_2_, as evident by the formation of a band at 2185 cm^−1^ (**Figure** **2**) that is identical to that observed under Ar (Figure 1). We hypothesize that the reaction between BTDN^•−^ and CO_2_ occurs at longer timescales than those of the CV and IR‐SEC experiments. This is evident by bulk electrolysis experiments monitored by UV–vis where BTDN^•−^ reacts with CO_2_ over the course of 30–60 min (Figures S7 and S8, S16–S18, Supporting Information).

Further reduction of BTDN^•−^ in the presence of CO_2_ results in transient formation of BTDN^2−^, with concomitant depletion of the band at 2342 cm^−1^ attributed to dissolved CO_2_. In addition, a new band at 2142 cm^−1^ is initially formed and rapidly depleted at more negative potential, this is suggestive of a transiently formed species by the reaction between BTDN^2−^ and CO_2_. At more negative potentials, an additional band at 2214 cm^−1^ is formed. At the same time, there are bands in the C—O stretching region being formed at 1645 and 1684 cm^−1^ (Figures 2 and S9–S15, Supporting Information).

During the reoxidation scan, partial recovery of BTDN^•−^ is observable at the same potential region corresponding to reoxidation of BTDN^2−^ to BTDN^•−^. This process appears to have slower kinetics compared to that observed under Ar atmosphere. At the same time, there is a slow decrease in intensity of the bands at 2214–1645 and 1684 cm^−1^ along with a slow increase in the intensity of the 2342 cm^−1^ band. These observations suggest that the reaction between BTDN^2−^ and CO_2_ may be reversible. An oxidation peak is observable at more positive potential (−1.04 V) and this corresponds to oxidation of BTDN^•−^, verified by the depletion of the corresponding band at 2185 cm^−1^.

**Figure 3:**
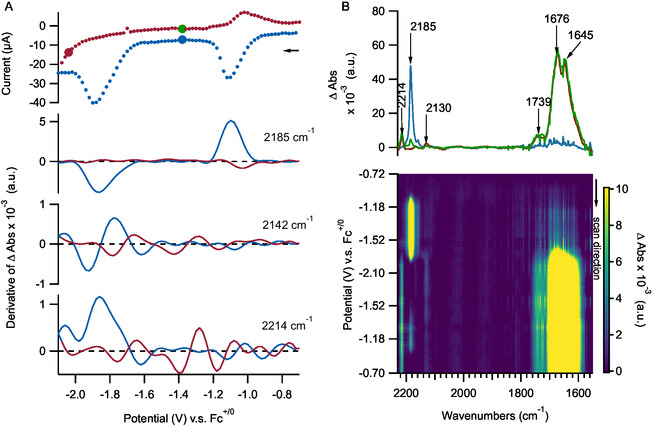
IR‐SEC of 2 mM solution of BTDN in acetonitrile with 0.2 M TBAPF_6_ supporting electrolyte under 3 bar 100% CO_2_. The potential step is 20 mV and the average scan rate is 15 mV s^−1^. A) Top panel: current–voltage response during IR‐SEC experiment. Bottom panels: derivative of peak intensity of ν(CN) bands as a function of applied potential. Blue traces indicate cathodic scans, red traces indicate anodic scans. B) Top panel: representative IR spectra recorded during IR‐SEC where the applied potential is color coded based on color of each spectrum matching the large circular symbol in panel A. Bottom panel: 2D image plot of IR spectra as function of applied potential. The false‐color image on the bottom right panel is capped at Δ abs value of 10 × 10^−3^ to aid visualization of bands with weaker intensity.

At higher CO_2_ partial pressure (100% CO_2_, 3 bar), reduction of BTDN follows a similar trend. There is no observable reaction between BTDN^•−^ and CO_2_ (**Figure** **3**). Upon further reduction of BTDN^•−^ at the second reduction wave, there is no observable BTDN^2−^ intermediate at 2111 cm^−1^ suggesting a higher rate for the reaction between BTDN^2−^ and CO_2_ when the concentration of CO_2_ is in large excess. Similarly, the band at 2142 cm^−1^ is not observable, consistent with assignment of this band to an intermediate species formed by the reaction between BTDN^2−^ and CO_2_. A new band at 2130 cm^−1^ is observable, suggestive of a new species formed under higher equivalence of CO_2_. The band at 2214 cm^−1^ is observable under excess of CO_2_, and the ratio of intensity between the 2214 and the 2185 cm^−1^ band (formed after the first reduction wave) is comparable to that observed under very low CO_2_ partial pressure (0.8% CO_2_, 2 bar). This suggests that the 2214 cm^−1^ band may be attributed to a side product without reaction with CO_2_, as the formation of this species does not appear to be influenced by CO_2_ concentration. At the C—O stretching region, there are additional bands formed at 1739 cm^−1^ and an apparent peak at 1676 cm^−1^. Notably, the relative intensities of the bands in the 1600–1700 cm^−1^ region are very distinct between low and high CO_2_ partial pressures, suggestive of multiple carbonyl species formed.

The ratio of peak reduction current *i*_p2_/*i*_p1_ between low and high CO_2_ partial pressure in IR‐SEC experiments (Figures 2 and 3) is not significantly different, indicating little dependency on CO_2_ concentration. This suggests that the reaction between BTDN^2−^ and CO_2_ is not likely to be catalytic.

**Figure 4:**
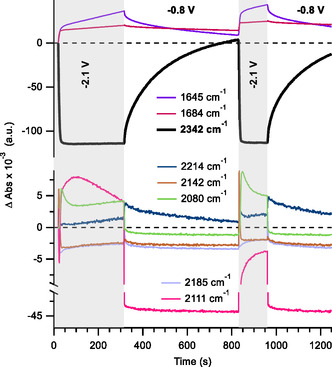
Relative intensity of IR band at 2342 cm^−1^ (highlight in bold) corresponding to free CO_2_ in solution during two cycles of reduction (−2.1 V vs. Fc^0/+^) and reoxidation (−0.8 V vs. Fc^0/+^) of BTDN during potential step IR‐SEC experiment, with 2 mM BTDN, 0.2 M TBAPF_6_ in acetonitrile. The gas composition is 2 bar of 0.8% CO_2_, corresponding to CO_2_:BTDN ratio of 2.16:1.0. The Δ Abs is calculated using the first spectra collected at −2.1 V; hence, formation of bands at 2185 and 2111 cm^−1^ during reoxidation appears as negative absorbance.

To gain additional insight into the reaction between BTDN^2−^ and CO_2_, potential step SEC experiments were carried out where BTDN is cycled between −2.1 and −0.8 V under low CO_2_ partial pressure (0.8% CO_2_,1.5 bar) (**Figure** **4**). The band at 2142 cm^−1^ appears as a transient species. Intriguingly, an additional band at 2080 cm^−1^ is observable. The wavenumber of the 2080 cm^−1^ band is lower than that of BTDN^2−^ (2111 cm^−1^), suggesting that it corresponds to a further reduced species of a product between BTDN^2−^ and CO_2_. Since this species is observable in potential step experiments but not in scanning SEC experiment, it is likely that it is a reduction product of a transient species formed by reaction between BTDN^2−^ and CO_2_. Since there are no discernible additional redox peaks, the reduction potential of this transient species is likely to be slightly more negative than the reduction potential of BTDN^•−^. In scanning SEC experiments, this transient species undergoes further chemical reaction before the potential is scanned sufficiently negative to enable its reduction, thereby precluding its formation. A plausible candidate may be reduction of the transient species with ν(CN) stretching frequency at 2142 cm^−1^ to form a further reduced species with ν(CN) frequency at 2080 cm^−1^.

Analogous potential step experiment was carried out under high CO_2_ concentration (100% CO_2_, 3 bar), showing the presence of similar ν(CN) bands (see Figure S9, Supporting Information). A notable exception is the absence of the 2111 cm^−1^ band attributed to BTDN^2−^ and the band at 2080 cm^−1^. The absence of the 2080 cm^−1^ band under high CO_2_ concentration suggests that it can be attributed to a reduced BTDN species that reacts with low equivalence of CO_2_. An additional band at 2130 cm^−1^ is initially formed and decays relatively slowly.

In addition to the observed changes in the ν(CN) and ν(CO) region when the potential is scanned passing the second reduction wave under CO_2_ atmosphere, there are two noticeable negative peaks at 3542 and 3632 cm^−1^, which correspond to OH stretching modes that can be attributed to presence of residual water in the solvent (Figure S10, Supporting Information). This suggests protonation reactions of BTDN^2−^ by residual water, or more likely H_2_CO_3_ formed between CO_2_ and residual water. The product of BTDN^2−^ protonation, monitored via the ν(CN) band, will not have strong CO_2_ concentration dependence, which is consistent with the observed band at 2214 cm^−1^. This assignment is further supported by density functional theory (DFT) calculations shown below. Another possible protonation pathway from residual water to BDTN^2−^ is via protonation of a carboxylate group formed when CO_2_ is bound to reduced BTDN as the pKa of such carboxylate group is expected to be lower compared to water.

The conjugate base formed by this protonation reaction is expected to form bicarbonate and carbonate species with ν(CO) bands in the 1750–1550 cm^−1^ region. Comparison of ν(CO) bands observed in SEC experiments with reference spectrum of chemically generated bicarbonate and carbonate species under similar conditions (Figure S22, Supporting Information) suggests additional carbonyl species derived from reaction between CO_2_ and reduced BTDN are formed.

### 2.3 Reversibility of the CO_2_ Bound States

When examining reduction of BTDN under low CO_2_ concentration (0.8% CO_2_, 2 bar; 4.32 mM),^[^ ^25^ ^]^ we note that there is a gradual recovery of free CO_2_ during the reoxidation phase of IR‐SEC experiments (see Figure 2). The release of CO_2_ from the reduced BTDN‐CO_2_ species is examined in more detail by potential step SEC experiments (Figure 4) where BTDN is reduced at −2.1 V versus Fc^0/+^ and subsequently reoxidized at −0.8 V Fc^0/+^ for two cycles. The relative band intensity at 2342 cm^−1^ shows almost reversible uptake and release of free CO_2_ for two cycles. We note the very slow kinetics for release of CO_2_ (*k* ≈ 6 × 10^−3^ s^−1^). This is consistent with the observation from scanning SEC experiment (Figure 2) where there is no distinct reoxidation peak that can be attributed to oxidation of a reduced BTDN‐CO_2_ species, suggestive of slow oxidation kinetics. At present, it is not possible to distinguish between oxidation kinetic and CO_2_ release kinetic as the rate limiting step.

During CO_2_ release, concurrent with the change in band intensity at 2342 cm^−1^, there are similar changes in the ν(OH) modes at 3542 and 3632 cm^−1^. This indicates that the reversible binding of CO_2_ to reduced BTDN is coupled to a protonation reaction. We note that although reversible CO_2_ binding and release appear to be coupled to a protonation reaction, CO_2_ binding and release is not simply based on the CO_2_‐bicarbonate acid–base equilibrium. The formation of a band at 2214 cm^−1^, assigned as protonation product of BTDN^2−^ by residual water, does open a pathway for direct bicarbonate formation from CO_2_ with resulting hydroxide ion. However, at −0.8 V the intensity of the 2214 cm^−1^ band does not decrease (Figures 2 and 3), as such the observed reversible CO_2_ capture and release cannot be rationalized by a simple acid–base equilibrium due to protonation of BTDN^2−^. It is likely that the observed reversible protonation behavior is associated with protonation of a carboxylate group of reduced BTDN‐CO_2_. At the reduced state of BTDN at −2.1 V versus Fc^0/+^, the pKa of the carboxylate group is higher than water resulting in proton transfer from residual water. At −0.8 V versus Fc^0/+^, oxidation of the BTDN‐carboxylic acid molecule results in lowering of its pKa, resulting in proton transfer to either hydroxide or bicarbonate molecule. This redox‐induced shift in pKa of carboxylic residue has been previously reported with ferrocene carboxylic acid.^[^ ^26^ ^]^ We hypothesize that the reversible protonation behavior appears to be a consequence of formation of BTDN‐carboxylic acid molecules and presence of residual water molecules, rather than the cause of CO_2_ capture and release. Similarly, the formation of protonated BTDN^2−^ (2214 cm^−1^ band) appears to be a side reaction due to the presence of residual water molecules.

### 2.4 Computation of Possible CO_2_ Bound States

**Figure 5:**
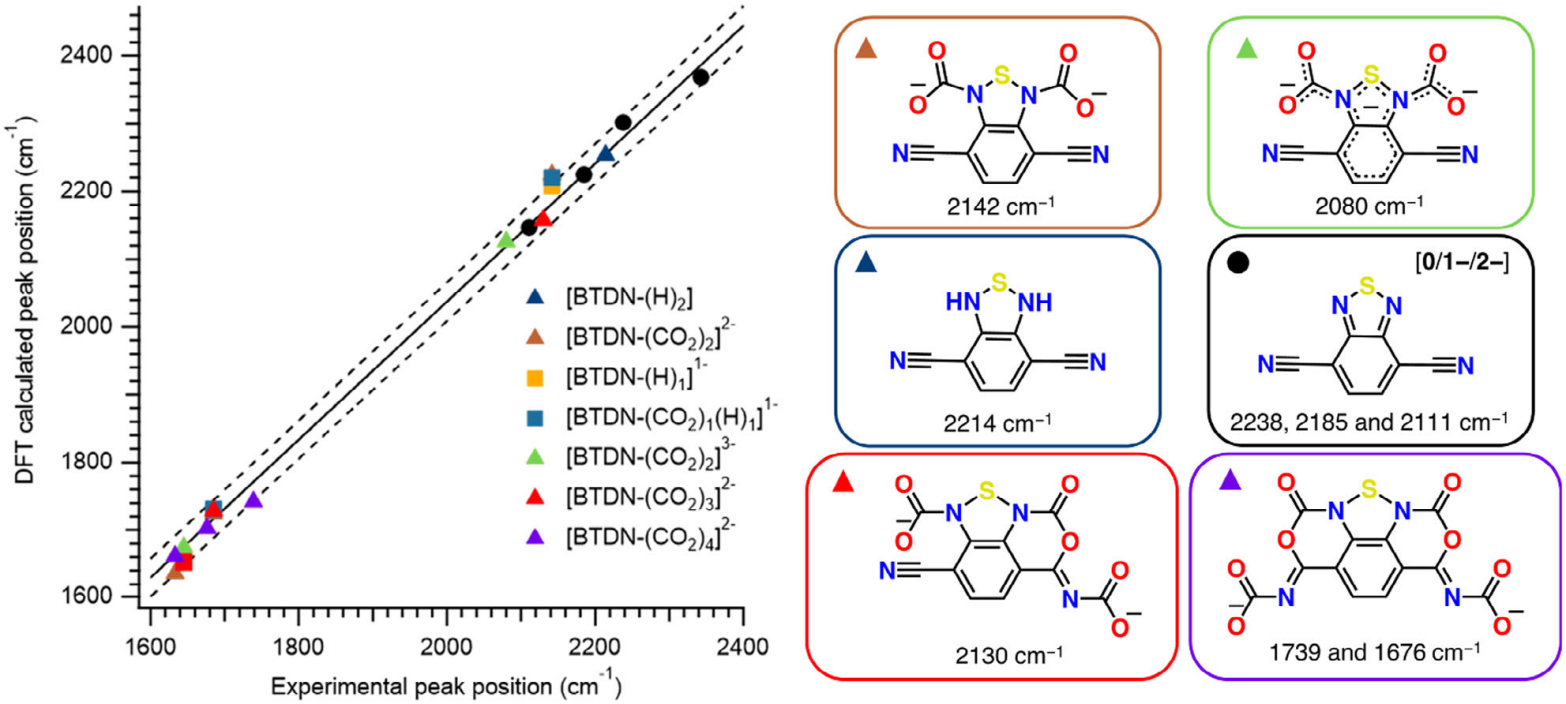
Comparison of the DFT‐calculated ν(CN) and ν(CO) bands at r2‐SCAN/def2‐TZVP(‐f) level of theory to experimental observations and their corresponding structures. Black points are CO_2_, BTDN, BTDN^•−^, and BTDN^2−^ species that are used to calibrate the DFT results. The calibration line is presented as solid black line while the 1 σ prediction band is plotted as dotted line. Experimental ν(CN) are listed below each structure.

DFT calculation^[^ ^27^, ^28^ ^]^ was performed to aid the assignment of the observed ν(CN) bands to reduced BTDN species and its reaction with CO_2_ and protons. The experimental wavenumbers of ν(CN) and ν(CO) bands of BTDN, BTDN^•−^, BTDN^2−^, and CO_2_ were used to calibrate the DFT‐calculated ν(CN) and ν(CO) bands presented in **Figure** **5**. As discussed earlier, the band at 2214 cm^−1^ is not likely to be a product between reduced BTDN and CO_2_ but rather a protonated product of BTDN^2−^. The predicted ν(CN) band of a [BTDN‐(H)_2_] is in very good agreement with experimental band at 2214 cm^−1^, thus supporting this assignment. The observed band at 2080 cm^−1^ can be assigned as a further reduced product between BTDN^2−^ and CO_2_; the predicted ν(CN) of a three‐electron reduced [BTDN‐(CO_2_)_2_]^3−^ is in good agreement with this experimentally observed band. The band at 2130 cm^−1^, observable under high CO_2_ concentration, can be assigned to [BTDN‐(CO_2_)_3_]^2−^ based on the good agreement between prediction and experiment. The transiently observed band at 2142 cm^−1^ can be modeled as either [BTDN‐(H)_1_]^1−^, [BTDN‐(CO_2_)_1_(H)_1_]^1−^, or [BTDN‐(CO_2_)_2_]^2−^. In light of the observation that it is not observable under high CO_2_ concentration conditions, assignment of the 2142 cm^−1^ band to [BTDN‐(CO_2_)_2_]^2−^ seems more plausible as it can react with additional equivalent of CO_2_ to form [BTDN‐(CO_2_)_3_]^2−^ and [BTDN‐(CO_2_)_4_]^2−^ species. For all DFT‐calculated species, the predicted ν(CO) bands are in a range that is in satisfactory agreement with experimental observations.

**Figure 6:**
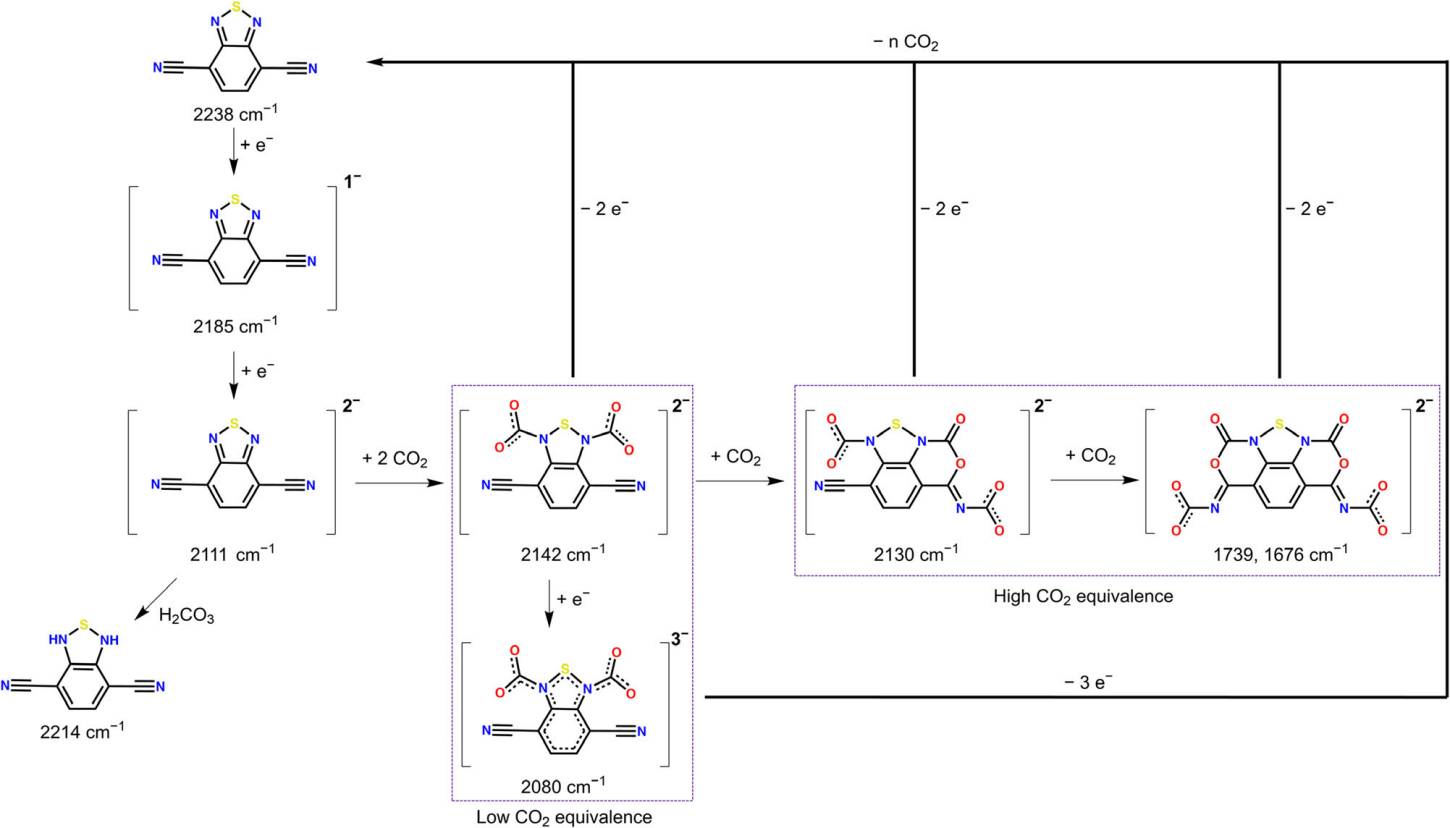
Proposed reaction pathways between reduced BTDN species and CO_2_. Black arrows indicate CO_2_ capture pathways, blue arrows indicate CO_2_ release.

Based on the IR‐SEC and DFT calculations, we propose the reaction pathways between BDTN^2−^ and CO_2_ presented in **Figure** **6**. BDTN undergoes two sequential one‐electron reductions, forming BTDN^•−^ and BDTN^2−^ successively. BTDN^•−^ does not react with CO_2_ within the timescale of the IR‐SEC experiments. BDTN^2−^ reacts with two equivalents of CO_2_ to form [BTDN‐(CO_2_)_2_]^2−^ (2142 cm^−1^) when partial pressure of CO_2_ is low. A further reduced product [BTDN‐(CO_2_)_2_]^3−^ (2080 cm^−1^) is observed only during potential step experiments, which suggest reduction of a non‐observable intermediate, most likely [BTDN‐(CO_2_)_1_]^2−^, before subsequent CO_2_ binding. At high CO_2_ partial pressure, [BTDN‐(CO_2_)_2_]^2−^ can react with additional CO_2_ to form [BTDN‐(CO_2_)_3_]^2−^ (2130 cm^−1^) and [BTDN‐(CO_2_)_4_]^2−^ (1739 cm^−1^). We also observed a side reaction of BTDN^2−^ with residual water to form [BTDN‐(H)_2_] (2142 cm^−1^). It is highly plausible that this protonation reaction is mediated by H_2_CO_3_, formed between H_2_O and CO_2_, due to the fact that [BTDN‐(H)_2_] is not formed in the presence of residual water without CO_2_. Most interestingly, we observed release of CO_2_ during oxidation of these [BTDN‐(CO_2_)_*x*_]^2/3−^ species, indicating redox‐induced CO_2_ capture and release; however, the kinetics of CO_2_ release appears to be much slower compared to CO_2_ binding. It is noteworthy to highlight two observations. First, our results indicate the possibility to capture up to four molecules of CO_2_ per BTDN molecule after reduction by two electrons. Second, CO_2_ capture and release can be achieved with dilute CO_2_ gas mixtures (0.8% CO_2_ in Ar). These observations suggest that BDTN can perform CO_2_ capture and release favorably compared to well‐known quinone‐based molecular systems.^[^ ^10^ ^]^

### 2.5 Electrochemical Reaction beyond the Second Redox Wave of BTDN

In addition of IR‐SEC experiments, we investigate the reduction of BTDN in the presence of CO_2_ with conventional CV experiments. As expected, the CV of BTDN under Ar shows reversible redox couple at −1.06 and −1.89 V versus Fc^0/+^ in MeCN.^[^ ^24^ ^]^

**Figure 7:**
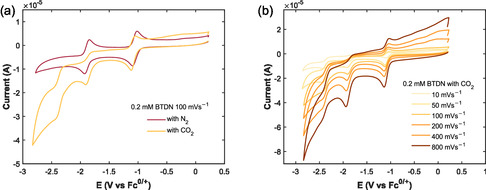
CVs of BTDN in the atmosphere of Ar and CO_2_. a) At 100 mV s^−1^, demonstrating the increase in current at the second reduction and the appearance of a third reduction wave. b) Scan rate dependence showing both differences in peak‐to‐peak current and the appearance of a wide oxidation peak starting after the oxidization of the first reduced species.

When the solution is saturated with CO_2_, there is a clear change in the current response (**Figures** **7** and S1–S6, Supporting Information). A new feature that arises under the CO_2_ atmosphere is a third reduction wave with an onset point around −2.1 V versus Fc^0/+^ reaching a plateau around −2.5 V versus Fc^0/+^. The current of this wave is about twice that of the first and second reduction waves of BTDN, the peak current ratio decreases at faster scan rates (Figure 7b and **Table** **1**).

**Table 1:** Peak currents and current ratios between the three reduction peaks with the third reduction at −2.4 V versus Fc^0/+^, at different scan rates for 0.2 mM BTDN saturated with CO_2_ in 0.1 M TBAPF_6_ in acetonitrile.

Scan rate [mV s^−1^]	*i*_pc1_ [μA]	*i*_pc2_ [μA]	*i*_pc3_ [μA]	*i*_pc1_/*i*_pc2_	*i*_pc3_/*i*_pc1_	*i*_pc3_/*i*_pc2_
10	3.20	3.73	6.17	0.86	1.93	1.65
50	7.97	6.29	11.33	1.26	1.42	1.80
100	9.23	8.85	13.54	1.04	1.47	1.53
200	12.32	12.22	16.13	1.01	1.31	1.32
400	15.02	17.74	17.49	0.85	1.16	0.99
800	21.25	24.14	19.30	0.88	0.91	0.80

The difference in peak current ratios between the scan rates shows that the reactions are quite slow or limited by mass transport of CO_2_. The comparatively large reductive current at the third reduction and the observation of only one oxidation peak show that multiple electrons have irreversibly been transferred to the presumptive BTDN‐CO_2_ adduct. The first reduction looks unperturbed when compared to the CV in an inert Ar atmosphere, this reduction is known to be a one‐electron reduction.^[^ ^24^ ^]^ Using the first reduction at slow scan rate as benchmark and comparing the reduction currents (Table 1), it can be seen that ≈2 electrons are transferred during the third reduction process at slow scan rates. The transfer of these electrons is not seen in the oxidative scan and hints at an irreversible reductive process taking place in the third reduction wave. At higher scan rates this ratio becomes much smaller, demonstrating that these reactions are limited either by the kinetics of the reaction or by mass transport.

The oxidative scan shows that the third and second reductions are mostly irreversible while the first reduction still retains some of its reversibility. However, the reversibility of the first reduction is also dependent on the scan rate and loses reversibility when scanning into the second and third reductions (Figures 7b and S6, Supporting Information). At scan rates higher than 10 mVs^−1^, a new oxidation peak was observed; this oxidation appears to be relatively fast compared to the other redox processes of BTDN with CO_2_. The signal is wide and undefined but occurs in the range of −0.20–0.45 V versus Fc^0/+^.

**Figure 8:**
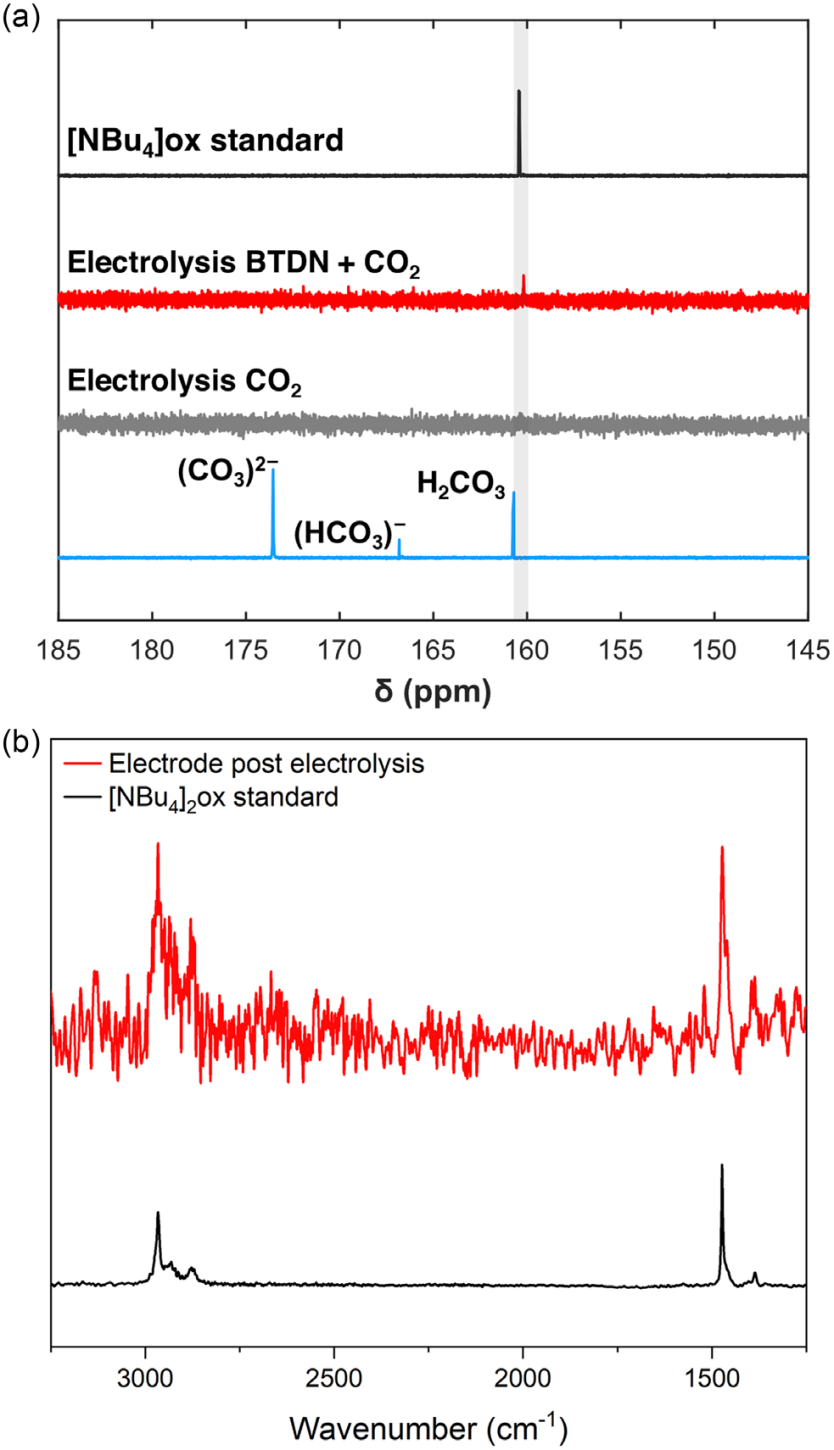
a) ^13^C NMR spectra of tetrabuthylammonium oxalate standard (black trace), the mixture of 1 mM BTDN saturated with CO_2_ in 0.1 M TBAPF_6_ in MeCN after 2 h electrolysis at −2.44 V versus Fc^0/+^(red trace), the saturated solution of CO_2_ in 0.1 M TBAPF_6_ 2 h electrolysis at −2.44 V versus Fc^0/+^ in MeCN (gray trace), and a solution of TBAOH in MeCN that was bubbled with CO_2_ for 12 h (light blue trace). b) Comparison of the ATR‐IR spectra of solid tetrabuthylammonium oxalate standard and the electrode surface after 2 h electrolysis of 1 mM BTDN and CO_2_ under the same conditions as described above.

Bulk electrolysis was carried out in an attempt to characterize the formation of CO_2_‐derived products at the potential of the third reduction of BTDN under CO_2_ atmosphere (−2.44 V vs. Fc^0/+^, see Figure S19, Supporting Information). Formation of bicarbonate and carbonate is well known in the CO_2_ reduction field.^[^ ^29^, ^30^ ^]^ However, comparison to the IR spectra of prepared salts (Figure S22, Supporting Information) suggested the formation of additional products. A signal with *δ* = 160 ppm is seen in the ^13^C NMR spectra of the electrolysis mixture (**Figure** **8**a); this supports the formation of oxalate or bicarbonate during the electrolysis.^[^ ^31^ ^]^ Moreover, since we used dry solvent in which only trace water can be present, bicarbonate should not be considered as a main product with very strong signal in NMR. For comparison, we bubbled a solution of tetrabuthylammonium hydroxide with CO_2_ for 12 h and measured the ^13^C NMR (Figures 8 and S23, Supporting Information). This data are used to generate ^13^C NMR reference spectra for carbonic acid, bicarbonate, and carbonate. In Figure S24, Supporting Information, there is a small but noticeable shift between electrolysis sample and reference spectra of carbonic acid. The observed signals are assigned as follows; the peak at 161 ppm is assigned to carbonic acid, the peak at 165 ppm is assigned to bicarbonate, and finally the peak at 169 ppm is assigned to carbonate. The signals originate from the equilibrium of carbonic acid in traces of water. A similar trend for the chemical shift of carbonate and bicarbonate species in ^13^C NMR has been reported in the literature albeit this was reported in D_2_O solvent instead of deuterated acetonitrile^[^ ^32^ ^]^. The experimental data suggest that oxalate was formed during the electrolysis experiments.

In addition, the surface of the electrode was analyzed by attenuated total reflectance (ATR)‐IR after the electrolysis experiment; a band in the fingerprint region is seen at 1474 cm^−1^ (Figure 8b), this band matches that of chemically prepared tetrabuthylammonium oxalate. Both ^13^C NMR and ATR‐IR support the presence of oxalate in solution and at the surface of the electrode respectively. In addition, negligible amounts of CO (122 nmol of CO) were detected in the headspace after 2 h bulk electrolysis experiment with BTDN under CO_2_ atmosphere. In the absence of BTDN, there was no oxalate detected implying the inertness of glassy carbon working electrode under these conditions.

To evaluate if an electroactive film derived from BTDN and CO_2_ is formed during the electrolysis, the glassy rod electrode was taken out of the electrolysis mixture and put into a solution of fresh electrolyte. No redox processes were detected in a typical CV measurement (Figure S21, Supporting Information) suggesting that the formation of an electroactive film did not occur under our electrolysis conditions. In another experiment, the electrode was taken out of the solution after the electrolysis, polished, washed with fresh electrolyte, and introduced back into the final electrolysis mixture and a CV was recorded for the mixture saturated with CO_2_ (Figure S22, Supporting Information). From this measurement, it is possible to see that most of BTDN was converted during the electrolysis but a redox process is seen as an irreversible wave with an onset potential of −2.0 V versus Fc/Fc^+^. This indicates that an electroactive species derived from BTDN and CO_2_ is formed during the electrolysis. This species persists in solution after 2 h of electrolysis and is electroactive in the presence of CO_2_.

## 3 Conclusion

The electrochemical reaction between reduced BTDN and CO_2_ has been investigated at three different reduction potentials. The reaction between the BTDN and CO_2_ can take place at every reduced state of BTDN and the CO_2_ molecules bound to a single BTDN unit or react with the reduced BTDN for possible catalytic reduction reactions. CO_2_ can be reversibly captured in the form of BTDN‐CO_2_ adducts and released by oxidizing the species generated at the second reduction. Finally, even though the majority of the electrons from the reduced BTDN seem to go into binding the CO_2_, at its third reduction potential BTDN seems to be able to mainly reduce CO_2_ to oxalate. Though it was hard to quantify the oxalate produced in the system, but the experimental evidence shows that the system has potential to reduce CO_2_ into a product. Moreover, this electrochemical reaction could probably be improved by varying the conditions by for example adding proton donors or by modifying the structure of the BTDN compound toward application of CO_2_ electrocatalysis, which requires further studies.

## Conflict of Interest

The authors declare no conflict of interest.

## Supporting information

Supplementary Material

## Data Availability

The data that support the findings of this study are available from the corresponding author upon reasonable request.
