# Supplementary material for: Electrochemical CO2 Capture, Release, and Reduction by a Benzothiadiazole Molecule with Multiple Redox States
Source: ChemSusChem. 2025 Oct 16;18(23):e202501724. doi: 10.1002/cssc.202501724 (PMC12665878; doi:10.1002/cssc.202501724)
Supplement: Supplementary file 1 — Supplementary Material [file CSSC-18-e202501724-s001.pdf]

Electrochemical CO_2 capture, release and reduction by a benzothiadiazole molecule with multiple redox states

Martin Axelsson[a], Carlos Enrique Torres-Méndez[a], Mun Hon Cheah*[b] and Haining Tian*[a]

[a] Dr. M. Axelsson, C.E. Torres-Méndez, Prof. H. Tian
Physical Chemistry, Department of Chemistry-Ångström Laboratory
Uppsala University
Box 523, 75120, Uppsala, Sweden
E-mail: haining.tian@kemi.uu.se

[b] Dr. M. H. Cheah
Molecular Biomimetics, Department of Chemistry - Ångström Laboratory, Uppsala
Uppsala University
Box 523, 75120, Uppsala, Sweden
E-mail: michael.cheah@kemi.uu.se

Contents

Materials & Methods 2
Additional experimental data 4
References 19

Materials & Methods

Spectroscopic grade acetonitrile (MeCN) (Sigma Aldrich UvaSol) dried with 3 Å molecular sieves was used in this study, with 0.15 M recrystallized tetrabutylammonium hexafluorophosphate $TBAPF_6$ as supporting electrolyte in (spectro-)electrochemical experiments. Other solvents and chemicals were purchased from Sigma-Aldrich and used as received unless stated otherwise.

Cyclic voltammetry

All cyclic voltammetry not coupled to spectroscopy was performed in a ∅ 2.5 x 5 cm cylindrical glass cell with an AUTOLAB PGSTAT302N potentiostat. As a working electrode ∅ 3 mm glassy carbon (GC) disk electrode was used and polished with 0.05 μM Al_2O_3 particle paste in-between measurements, as a counter electrode a Pt wire was used separated from the bulk solution with a porous glass frit. As a reference electrode, a silver-wire (Ag/Ag^+) pseudo reference was used in MeCN with the electrolyte in a glass tube connected to the solution with a porous Vycore frit, the potential is then confirmed with ferrocene as an internal standard. The gas atmosphere was changed by, first saturating the gas with the solvent in a pre-bubbler with the same solvent as in the sample, and then flowing the gas through the sample for 20 minutes.

Bulk electrolysis

Bulk electrolysis was performed in two different setups. The generation of the $BTDN^{\bullet-}$ radical anion from BTDN was performed in an open H-Cell inside of an Ar atmosphere glovebox with BTDN in both THF and MeCN. Glassy carbon foam connected by glassy carbon rods was used as both working and counter electrode. The silver-wire (Ag/Ag+) pseudoreference was used as reference electrode. The experiment was run after the first reduction potential at −1.1 V vs Ag/Ag^+ with stirring for 3h or until the current reached a plateau.

The second bulk electrolysis setup was used to explore the reduction of BTDN under CO2 atmosphere at −2.44 V vs $Fc^{0/+}$ and identify the products formed by IR and ^{13}C NMR. This setup consists of a ∅ 2.5 x 5 cm cylindrical glass cell sealed with a rubber stopper with holes for the electrodes. A ∅ 5 mm x 10 cm GC rod was used as the working electrode. Both the Ag/Ag^+ reference electrode and the Pt-wire counter electrode were separated from the primary solution in ∅ 5 mm x 10 cm chambers with only electrolyte, which is isolated from the working compartment by a porous glass frit. In this experiment 5 mL of a 1 mM BTDN solution in acetonitrile were employed. Solution was bubbled with N_2 for 30 minutes, then bubbled with CO_2 for another 30 minutes in order to achieve CO_2 saturation.

Gas analysis

Gas analysis from the bulk electrolysis was performed by sampling solution headspace with a 100 μL Hamilton® syringe and then measured in a Thermo Scientific Trace 1300 gas chromatograph.

Analysis of liquid reaction mixtures

The solutions from bulk electrolysis experiments were studied using ^{1}H NMR, ^{13}C NMR, as well as FTIR. The NMR was measured in CD_3CN solvent and a Jeol Resonance 400 MHz spectrometer at 293 K. The FTIR was measured with the Bruker Veretex 70V spectrometer with an MCT detector.

Preparation of tetrabuthylammonium oxalate

This salt was prepared as internal standard to be used in ^{1}H NMR experiments for comparison with the final mixtures of bulk electrolysis experiments. A method from the literature was used for the synthesis.[1] In short, a round bottom flask was loaded with 2.0 g (2.5 mmol) of $[NBu_4]OH \cdot 30H_2O$ and 112.5 mg (1.25 mmol) of oxalic acid were dissolved in 25 mL of MeOH and stirred overnight. The solvent excess was removed by rotary evaporation, a white solid was isolated. The product was dried under vacuum for 12 hours without further purification. ^{1}H NMR (400MHz, CD_3CN): δ 3.17 (m, 16H), 1.59 (m, 16H), 1.34 (sext, *J*= 7.4 Hz, 16H), 0.96 (t, *J* = 7.3 Hz, 24H).

UV-vis SEC

UV-vis spectroelectrochemistry (SEC) was performed in a 1x1 cm cuvette with a custom lid to accommodate the electrodes. As a working electrode a custom 1.0 x 0.1 x 3.0 cm glassy carbon foam electrode with three graphite rods and copper tape as an electric contact. As a counter electrode a ∅ 1 mm Pt wire was used, the previously described silver-wire (Ag/Ag^+) pseudo reference was used. The spectroscopy was performed using an Agilent 8453 diode array, either directly or using a glass fibre connection to a glove box.

IR SEC

Infrared spectroscopy spectro-electro chemistry (IR SEC) was performed in a Bruker Vertex 70V spectrometer with an MCT detector, interfaced to a Biologic SP300 potentiostat and a custom SEC cell was employed.[2] IR SEC is measured in reflectance mode; a thin solution film is trapped between the reflective surface of a ∅ 3 mm GC disk electrode and CaF_2 window. The solution film is in contact with the bulk solution where the reference and counter electrode are placed. The reference electrode here was Ag/AgCl(s) wire pseudo reference in the same electrolyte as the bulk solution separated by a frit made of a 4 Å molecular sieve and the counter electrode is a ∅ 2 mm x 30 mm graphite rod. The cell was connected to a gas cylinder to allow measurements under difference gas composition and pressures.

With this setup, two types of experiments were performed; in the first type a stair case CV scan with 20 mV steps are conducted while spectra were collected at each potential. The second type was a large potential step experiment where consecutive spectra collection during the potential step allows collection of time series of spectra under set potential.

Preparation of TBA Salts

Starting material: tetrabutylammonium hydroxide triacontahydrate (TBAOH).

Synthesis of tetrabutylammonium hydrogencarbonate ($TBAHCO_3$)[3]: In a round bottom flask, 2.0 g (2.5mmol) of TBAOH was dissolved in 25 mL of MeOH and bubbled with CO_2 gas for 24 hours at room temperature. The solvent excess was removed by rotary evaporation and a white solid was obtained. The product was dried under vacuum for 12 hours without further purification. 1H NMR (400 MHz, D2O): δ 3.17 (m, 8H), 1.63 (m, 8H), 1.34 (sext, J= 7.4 Hz, 8H), 0.92 (t, J = 7.4 Hz, 12H).

Synthesis of tetrabutylammonium carbonate ($(TBA)_2CO_3$)[3]: In a round bottom flask, 0.89 g (2.9 mmol) of $TBAHCO_3$ and 2.33 g of TBAOH (2.9 mmol) were dissolved in 25 mL of MeOH and stirred overnight. The solvent excess was removed by rotary evaporation and a yellow solid was obtained. The product was dried under vacuum for 12 hours without further purification. 1H NMR (400 MHz, D2O): δ 3.17 (m, 16H), 1.62 (m, 16H), 1.33 (sext, J= 7.4 Hz, 16H), 0.92 (t, J = 7.4 Hz, 24H).

Additional experimental data

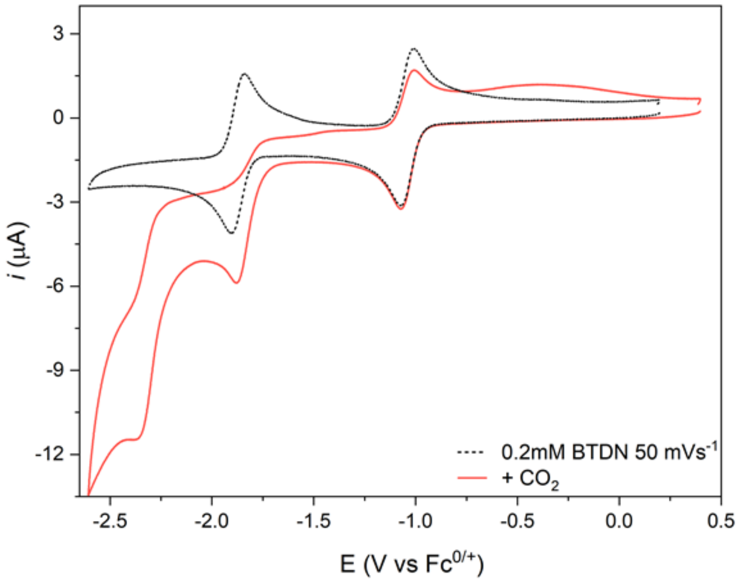

Figure S1. The CVs of 0.2 mM BTDN in Ar and CO_2 atmosphere at 50 mVs^{-1}.

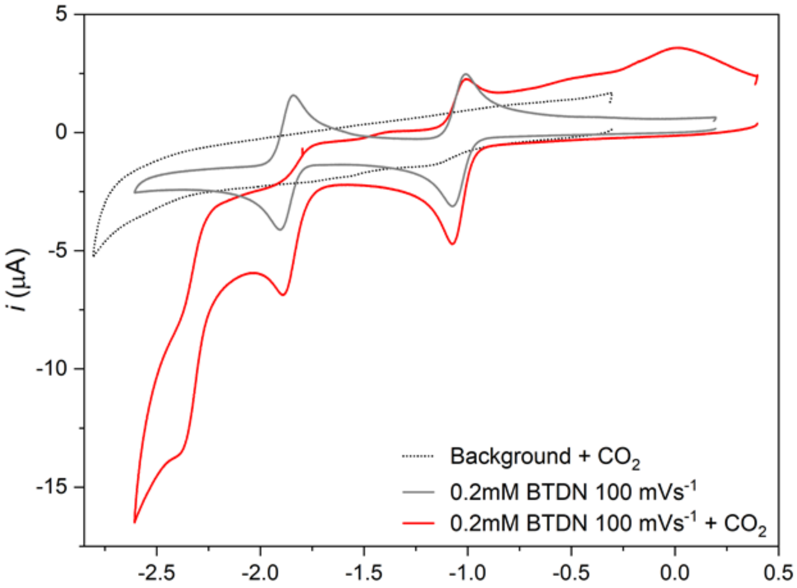

Figure S2. The CVs of 0.2 mM BTDN in Ar and CO_2 atmosphere at 100 mVs^{-1} and background signal under CO_2 atmosphere.

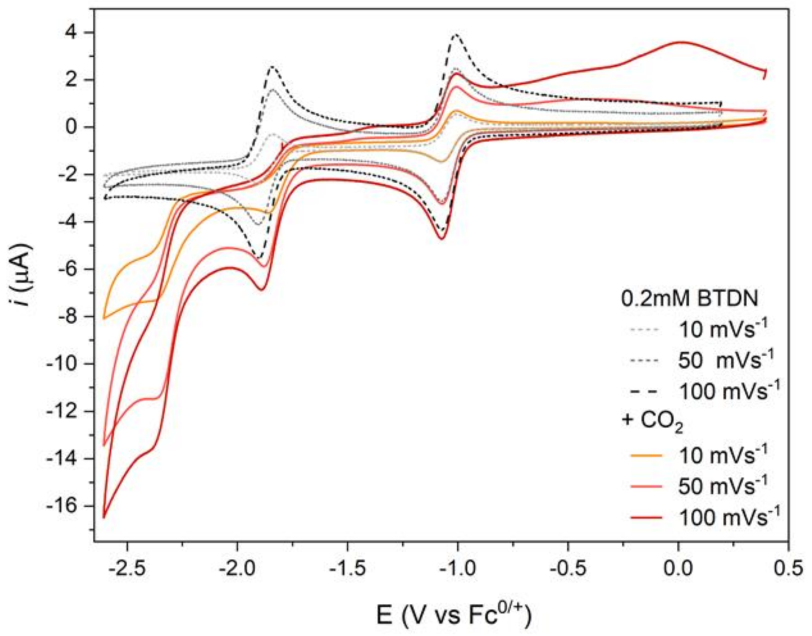

Figure S3. The CVs of 0.2 mM BTDN in Ar and CO_2 atmosphere at 10, 50 and 100 mVs^{-1}.

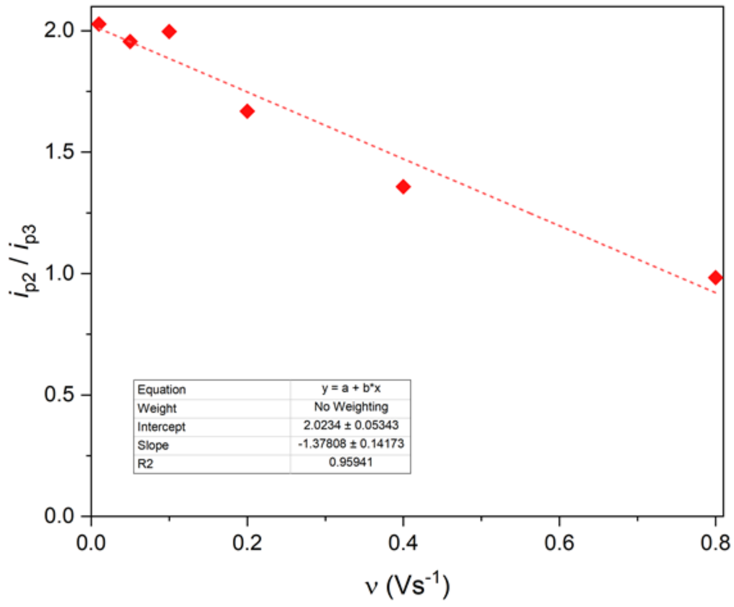

Figure S4. The peak current ratio between the second and third reduction over scan rates as well as a linear fit to the behavior.

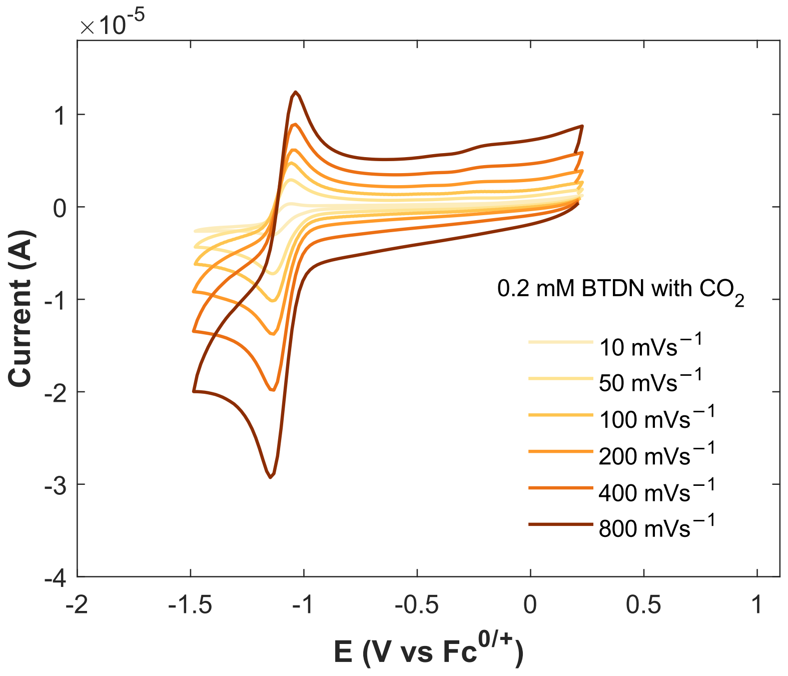

Figure S5. Scan rate dependance of BTDN under CO_2 atmosphere. Scanning up to the first reduction of BTDN.

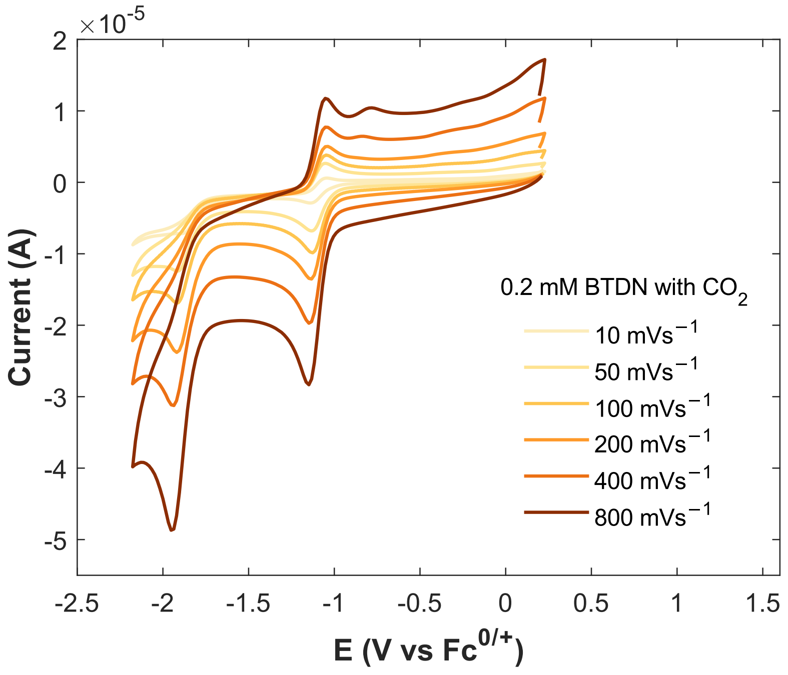

Figure S6. Scan rate dependence of BTDN under CO_2 atmosphere. Scanning up to the second reduction of BTDN.

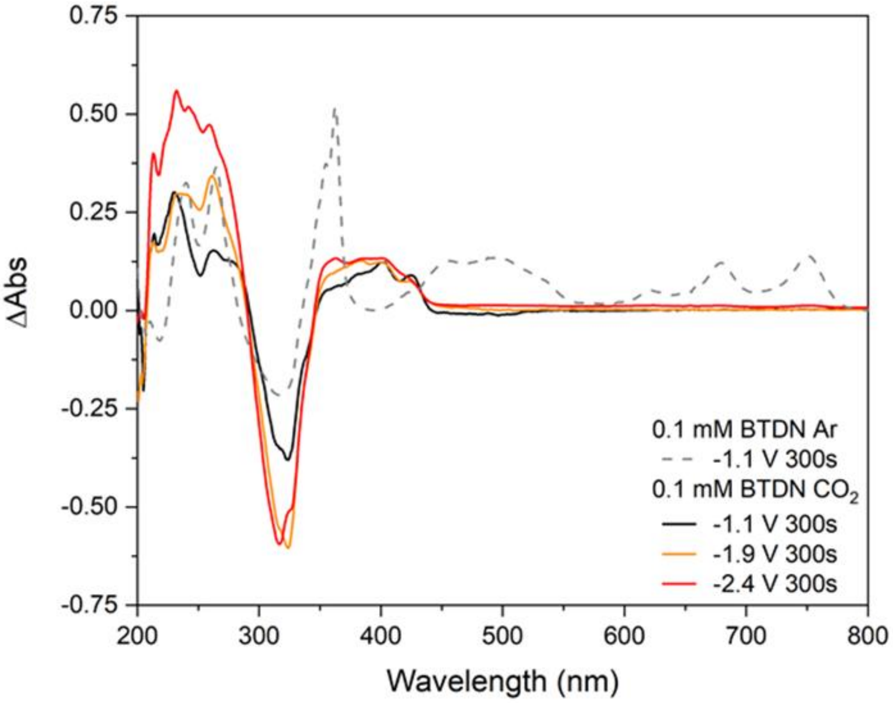

Figure S7. SEC UV-Vis of 0.1 mM BTDN at three reduction potentials, −1.1 V, −1.9 V, and −2.4 V vs $Fc^{0/+}$ for 300 s each in presence of CO_2 as well as −1.1 V vs $Fc^{0/+}$ spectrum in Ar.

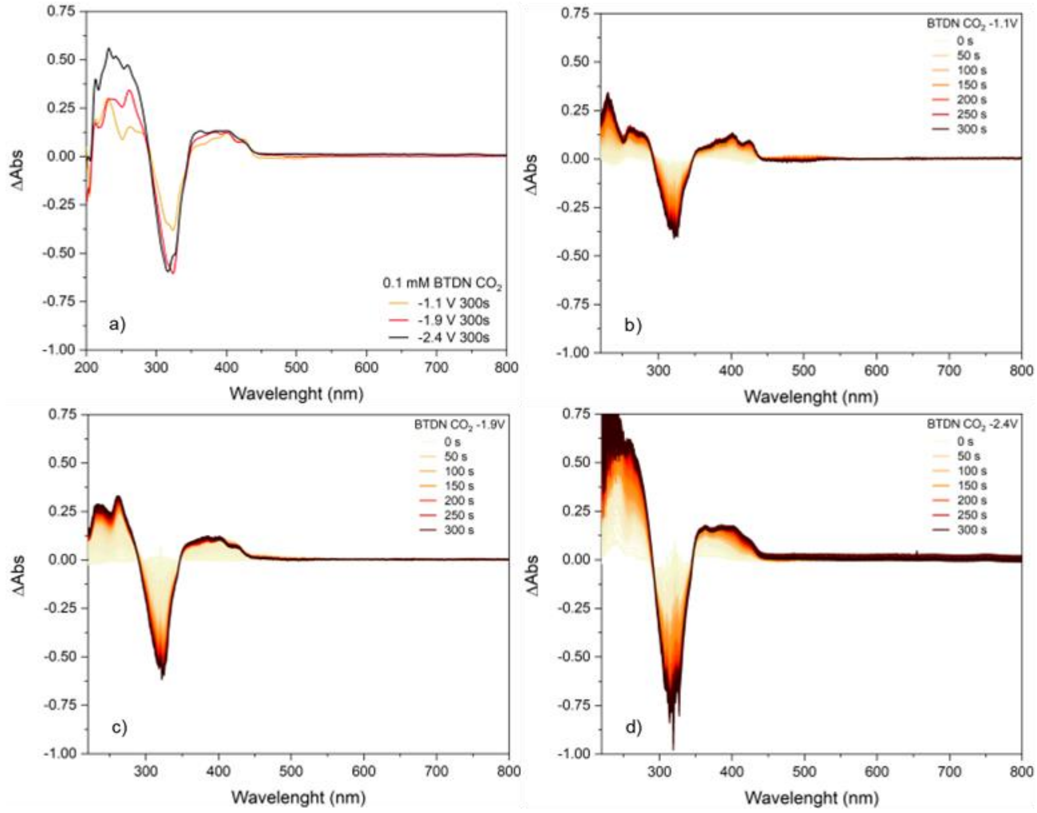

Figure S8. Showing the data from the SEC UV-Vis of 0.1 mM BTDN at three reduction potentials, b) −1.1 V, c) −1.9 V, and d) −2.4 V vs $Fc^{0/+}$ for 300 s, all going from light orange to dark red over the duration of the experiment. As well as a) the comparison of the spectra of

the reduced species after 300 s. All of the species show a bleach in the 323 nm range where BTDN absorbs as well as new spectral bands both in the 400-450 nm and the 200-300 nm range but with different shapes and amplitudes.

To investigate the different species that form at each reduction event, spectroelectrochemistry (SEC) was utilized, in both the spectral regions of UV-Vis and IR. In the UV-Vis SEC data (Figures S7, S8 and S16-S18) can be seen, and it is clear that a different reaction with CO_2 occurs at each reduction. Even at the first reduction, there are obvious spectral differences compared to well-known singly reduced BTDN radical anion ($BTDN^{\bullet-}$, Figure 2).[4–8] This reactivity was not obvious from CV experiments alone and underline the importance SEC studies. The difference between the CV and SEC is likely the timescale of the experiments, showing that the reaction is quite slow since the SEC experiment is performed at the set potential for a longer time. Both the second and third reduction show the growth of new features expanding into the visible spectra. Both species are clearly different from the singly reduced state and different from the $BTDN^{2-}$ species forming in the absence of CO_2.[4] The UV-Vis SEC data also shows that there are different reactions occurring at all three reduction potentials with clear differences in the features in both the 350-450 nm range and 200-300 nm range.

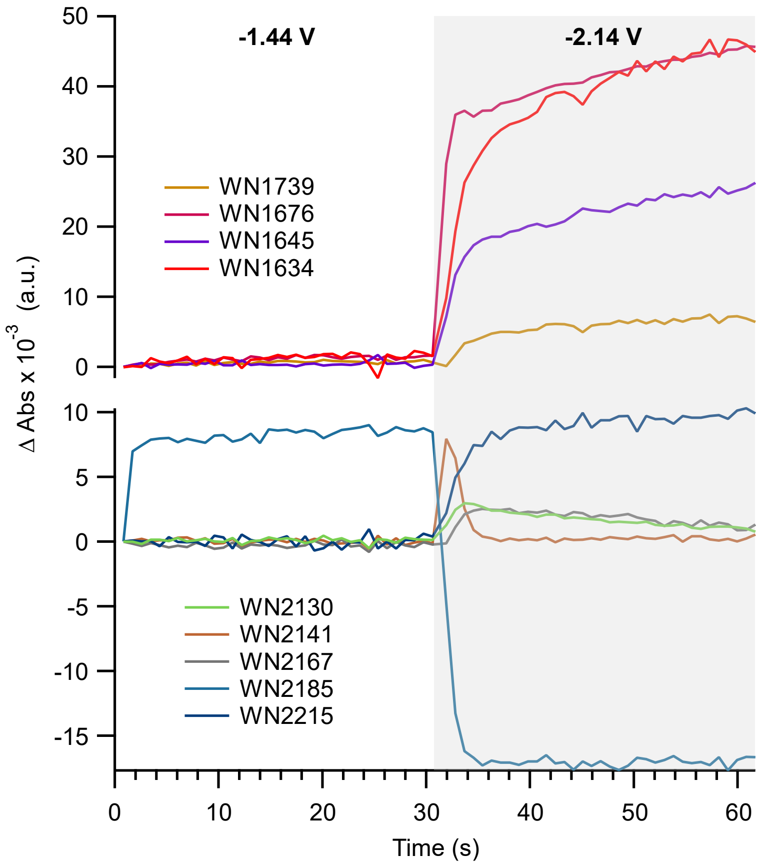

Figure S9. Relative intensity of IR bands (WN: wavenumber) solution of BTDN during potential step IR-SEC experiment, with 2 mM BTDN, 0.2 M $TBAPF_6$ in acetonitrile. The gas composition is 3 bar of 100% CO_2.

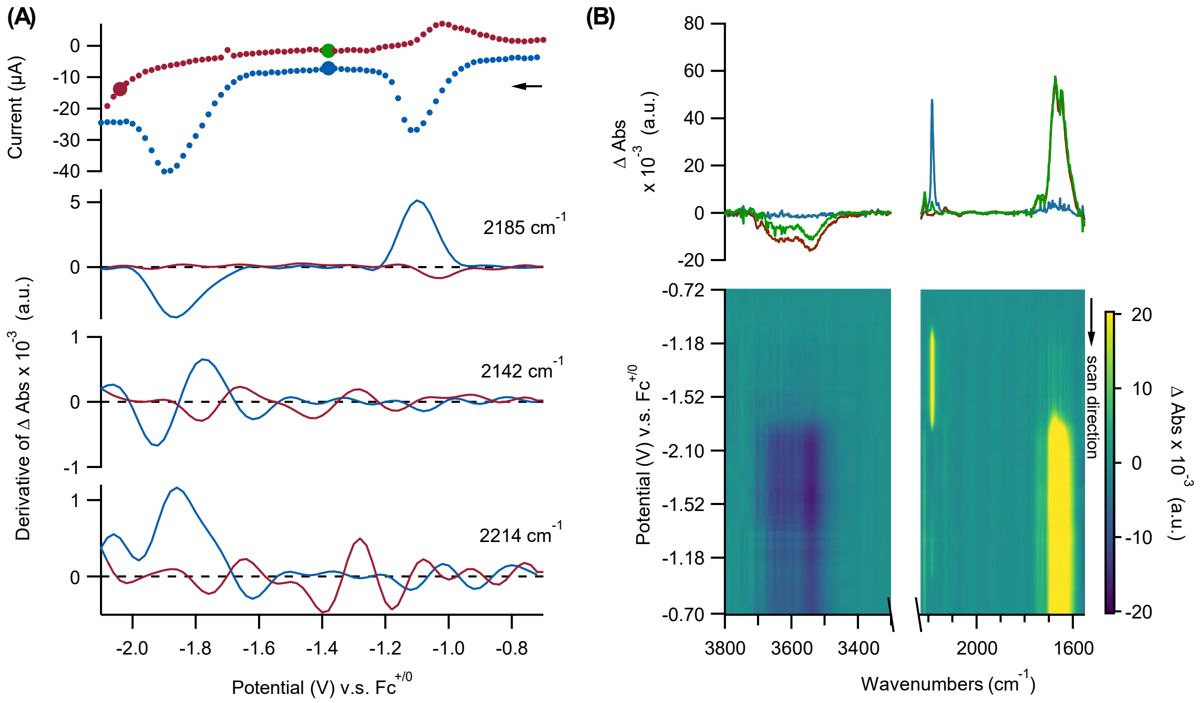

Figure S10. Expanded view of Figure 5, including the ν(OH) region, during IR-SEC of 2mM solution of BTDN in acetonitrile with 0.2 M $TBAPF_6$ supporting electrolyte under 3bar 100% CO_2. The potential step is 20 mV and the average scan rate is 15 mV/s.

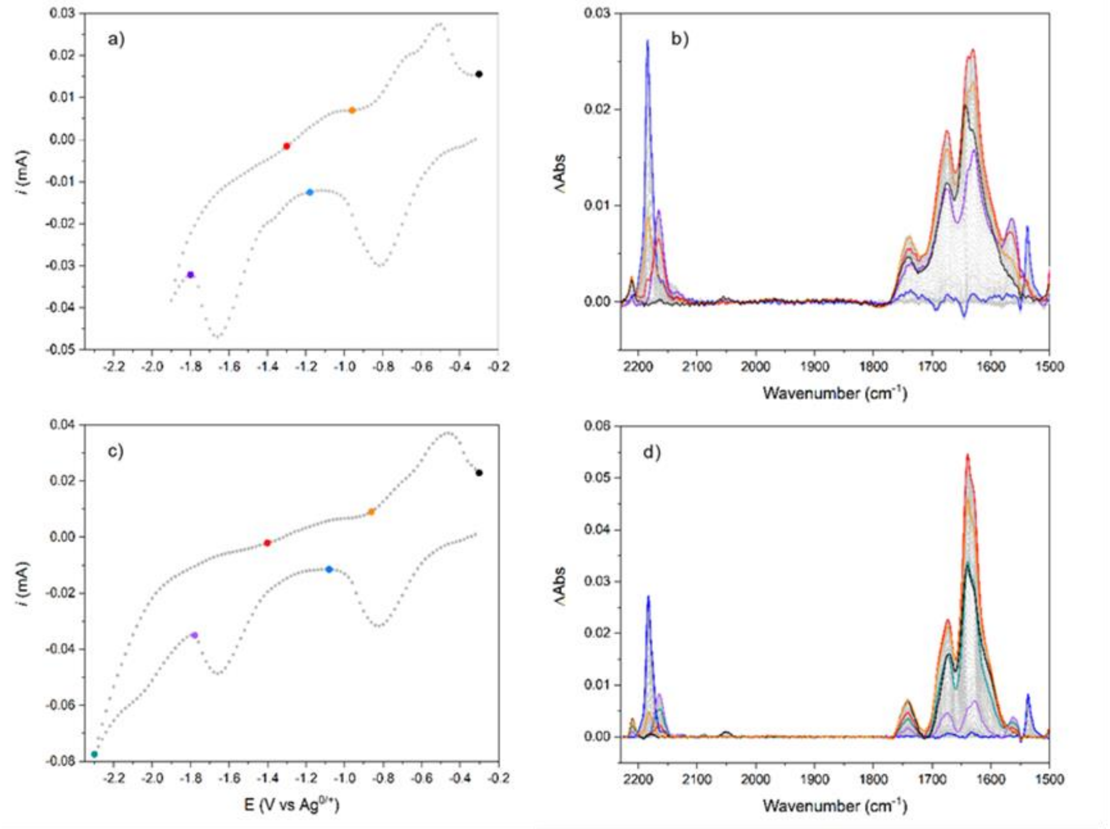

Figure S11. Showing the SEC IR measurements, five CVs and corresponding IR spectra with coloured spectra corresponding to specific points in the CVs. Stopping at the second reduction (a and b) shows a shift to a 2165 cm^{-1} C≡N vibration that arises together with peaks 1740 cm^{-1}, 1673 cm^{-1}, and 1630 cm^{-1} and the peaks in this region keeps growing even on the back scan, and a peak at 1564 cm^{-1} that starts to decline right away. When moving towards the third reduction (c and d) most of the same peaks appear but the 1639-1648 cm^{-1} peaks keep growing together for a longer duration. On the scan back the 1740 cm^{-1} peak keeps growing during the entire scan and the reappearance of the 2184 cm^{-1} BTDN$^{\bullet-}$ peak is much smaller.

Spectroscopical investigation of CO_2 bound states

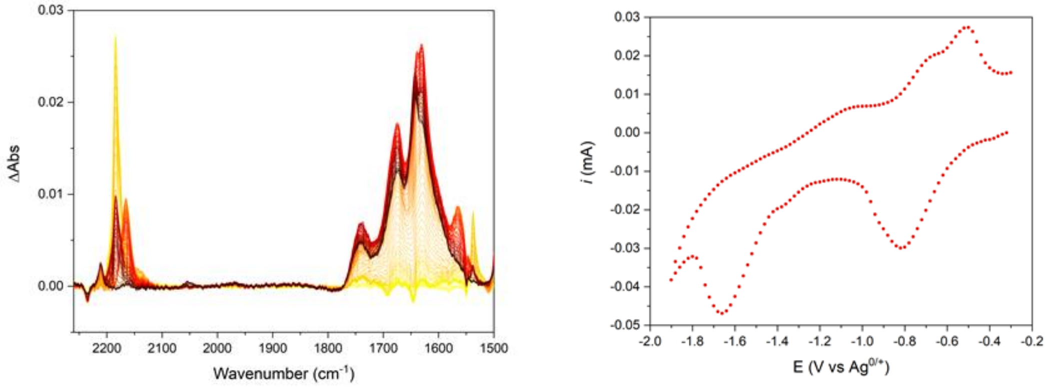

Figure S12. SEC IR of 5mM BTDN and 2 Bar CO_2 in THF scanning past second reduction at −1.9V.

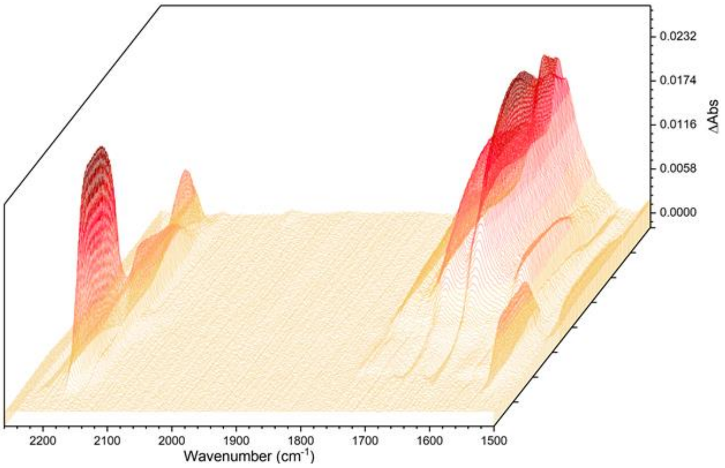

Figure S13. 3D plot of the SEC IR of 5mM BTDN and 2 Bar CO_2 in THF scanning past second reduction at −1.9V.

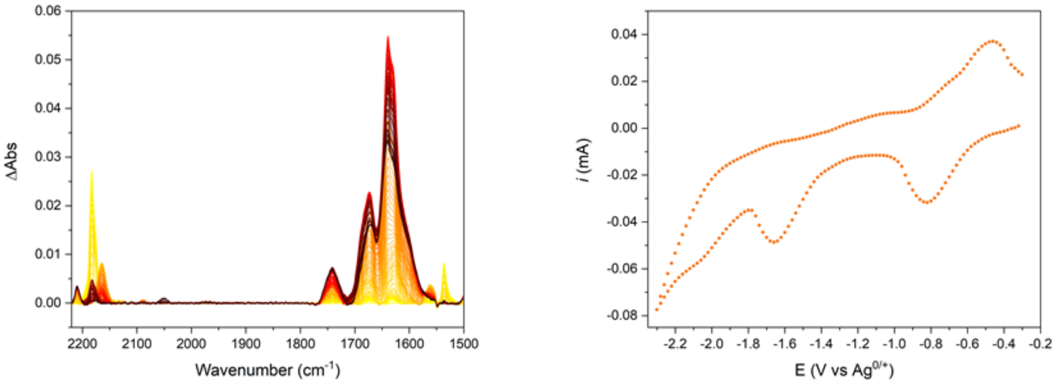

Figure S14. SEC IR of 5mM BTDN and 2 Bar CO_2 in THF scanning past the third reduction at −2.3V.

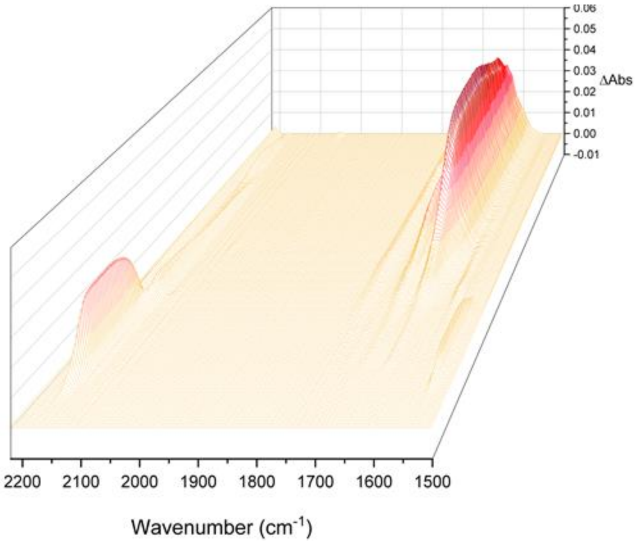

Figure S15. 3D plot of the SEC IR of 5mM BTDN and 2 Bar CO_2 in THF scanning past third reduction at −2.3V.

Control measurement with Ar inert atmosphere

The singly reduced species

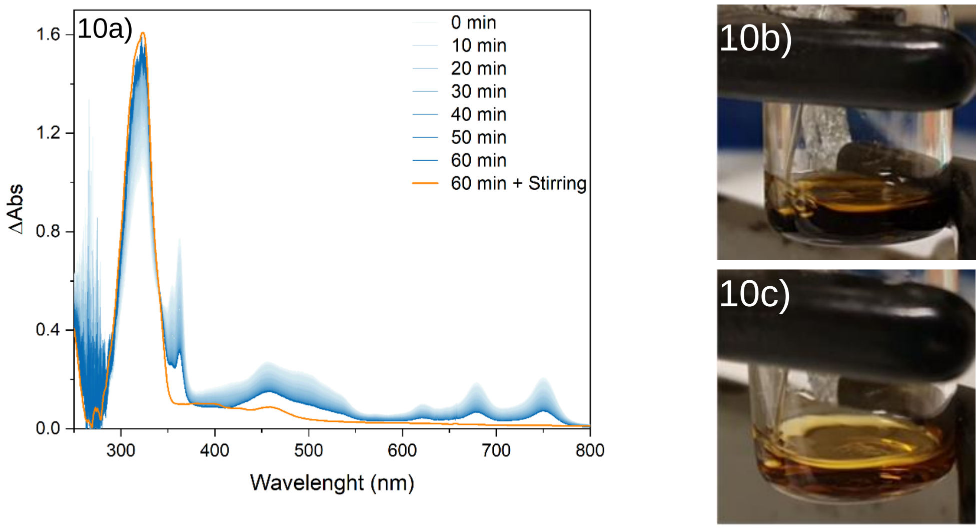

Figure S16. a) Showing the UV-Vis spectra of the $BTDN^{\cdot-}$ radical anion as it reacts with CO_2 by diffusing in solution. The final part of the reaction was achieved by stirring the cuvette. b) $BTDN^{\cdot-}$ solution bubbled with CO_2 gas corresponding to the initial min 0 spectrum and c) at the end of the reaction corresponding to the 60 min + stirring spectrum.

While it does not appear in the SEC IR experiments the $BTDN^{\cdot-}$ reaction with CO_2 is nonetheless interesting especially since it occurs at such comparatively low potentials. $BTDN^{\cdot-}$ is a very stable radical anion species and so can be regenerated at high concentrations in bulk electrolysis, this has previously been investigated both by us and others[4–8]. When this species is exposed to an atmosphere of CO_2 there is an obvious color change of the solution from a very dark brown to a clear orange color (Figure S16b). This color change can be followed spectroscopically both by flowing CO_2 into the headspace of a cuvette where the bleach of the anion species takes the main features as the largest new absorption at 323 nm corresponds well with the ground state BTDN transition (Figure S17). This new species also has a couple of new bands as a shoulder to the main BTDN transition these are the same bands that are visible in the SEC studies of the first reduction (Figure S18a).

To make sure that it is not O_2 leaking in and oxidizing the $BTDN^{\cdot-}$ back to BTDN we performed the same experiment in a cuvette with CO_2 saturated MeCN in the inert conditions of a glove box. In this experiment (Figure S16a), we see that $BTDN^{\cdot-}$ features very slowly start to decay over 60 minutes and finally after stirring the cuvette the same features appear here as well. On top of excluding O_2 as the reactive species it also tells us that this is a very slow reaction. This slow reaction even with very low concentrations of BTDN explains why this species is not clearly visible in the FTIR-SEC experiment where the BTDN concentration is much higher. The final spectrum is not exactly the same as the SEC UV-Vis experiment at the first reduction, as there is a new band at 470 nm (Figure S18). The large timescale difference between the experiments could be an explanation for this as a slower equilibrium would have time to establish itself in the longer time frame of the diffusion-based experiment.

The bound state is stable under Ar conditions but loses its color over time. The CO_2 can also be released by the reaction with an acid, we can both see this in IR that the peak of dissolved

CO_2 increases as the CO_2 bound state reacts (Figure S19). The same behavior can be seen with gas chromatography as CO_2 can be detected after the acid is added (Figure S20).

Table S1. Showing the integrated intensity of the CO_2 peaks from the gas chromatography experiment

Sample	Integrated area (pA min)	FWHM
Pre acid addition	98.64	0.1845
Post acid addition	1033.2841	0.2004

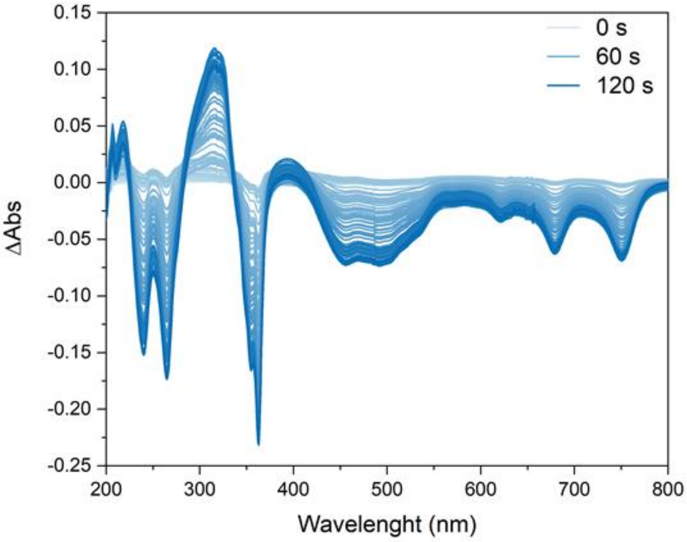

Figure S17. UV Vis of the transformation of BTDN$^{\cdot-}$ as it reacts with CO_2 that is flown through the headspace of the cuvette.

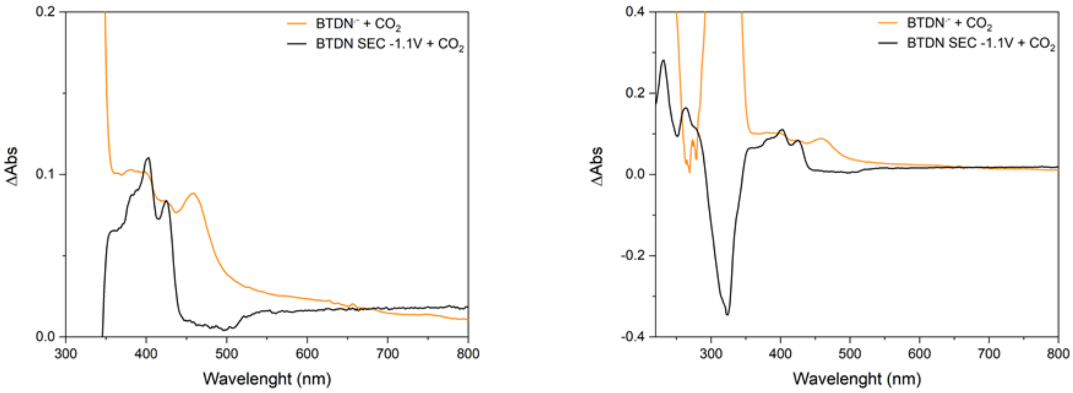

Figure S18. The comparison of the UV Vis SEC spectrum of BTDN with CO_2 (Black) and the reaction of BTDN$^{\cdot-}$ with saturated CO_2 solution (Orange).

Bulk electrolysis and post electrolysis electrochemistry

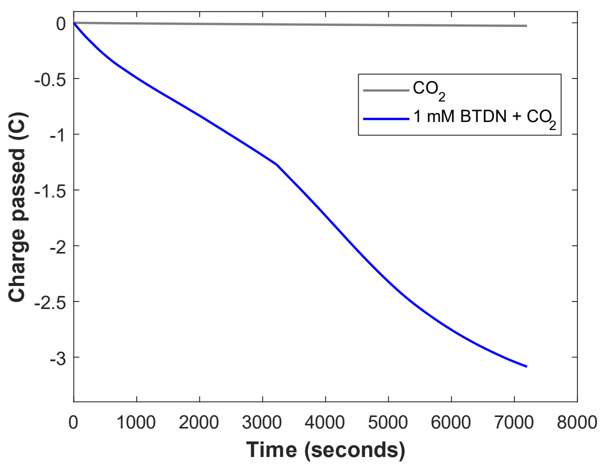

Figure S19. Charge passed in 2 hours of bulk electrolysis at −2.44 V vs $Fc^{0/+}$, mixture containing 0.1 M $TBAPF_6$ supporting electrolyte in MeCN.

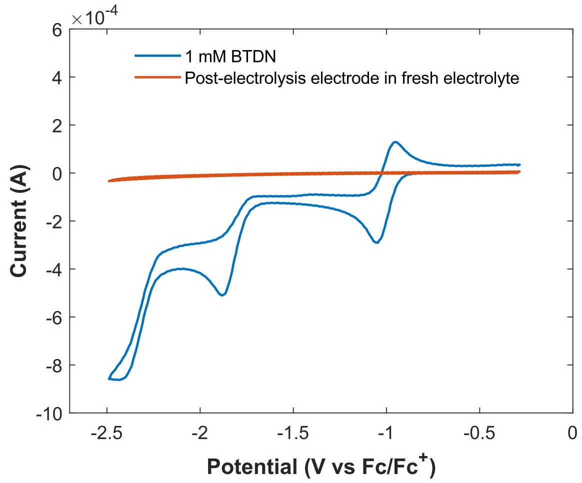

Figure S20. CV of fresh 1 mM BTDN in 0.1 M $TBAPF_6$ solution saturated with CO_2 and CV of the electrode taken into a fresh solution of 0.1 M $TBAPF_6$ in acetonitrile right after 2 hours of electrolysis with BTDN and CO_2.

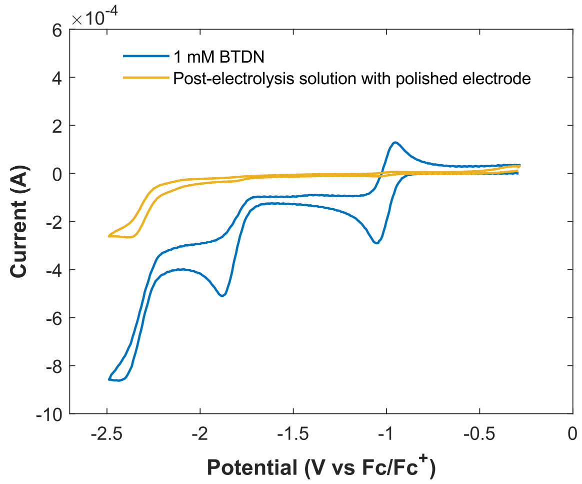

Figure S21. CV of fresh 1 mM BTDN in 0.1 M $TBAPF_6$ solution saturated with CO_2 and CV of the mixture obtained post-electrolysis (saturated with CO_2) with a polished electrode.

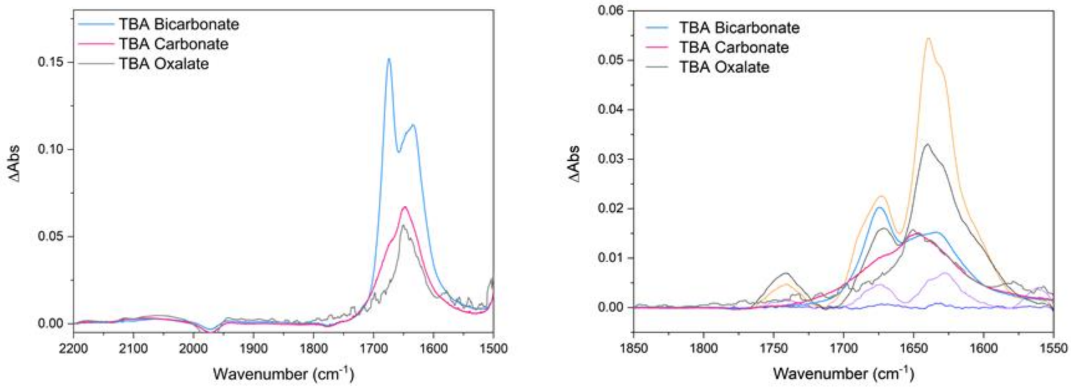

Figure S22. The IR spectra of TBA carbonate, TBA bicarbonate, and TBA oxalate both on their own as measured and adjusted in intensity to fit the spectra from the third reduction SEC IR CV experiment.

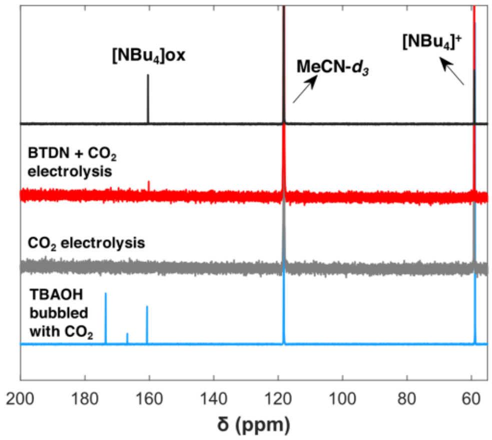

Figure S23. ^{13}C NMR spectra of tetrabuthylammonium oxalate standard, the mixture of 1 mM BTDN saturated with CO_2 in 0.1 M $TBAPF_6$ in MeCN after 2-hour electrolysis at −2.44 V v.s. $Fc^{0/+}$, the saturated solution of CO_2 in 0.1 M $TBAPF_6$ 2-hour electrolysis at −2.44 V v.s. $Fc^{0/+}$ in MeCN and a solution of TBAOH in MeCN that was bubbled with CO_2 for 12 hours.

DFT coordinates

BTDN

H	1.22977544162049	-0.00000000060987	3.07920798246906
C	0.70352061747067	0.00000000503248	2.13315405004628
C	-0.70352059773962	-0.00000000117230	2.13315405742746
H	-1.22977542054971	-0.00000000891672	3.07920800647354
C	-1.42680599612358	-0.00000000032554	0.95450855952694
C	-0.71747578522008	0.00000000088288	-0.27587511379276
C	0.71747576757358	0.00000000330793	-0.27587511082560
C	1.42680601689860	0.00000000787188	0.95450856186589
N	-1.25044265320179	-0.00000000242986	-1.50594775129185
N	1.25044259485704	-0.00000000043989	-1.50594776486927
S	-0.00000003759971	-0.00000000395317	-2.53011062820800
C	2.84682933045185	0.00000000105821	0.95532068806653
C	-2.84682930336033	0.00000000135728	0.95532065247441
N	-4.00354266370876	0.00000000287177	0.94961411642256

N	4.00354268863135	-0.00000000453509	0.94961419362302

BTDN^{1-}

H	1.22646603364149	0.00000000078232	3.10101913008849
C	0.68891809300175	0.00000000070932	2.16054682968648
C	-0.68891809299562	0.00000000053319	2.16054682824362
H	-1.22646603441567	0.00000000008799	3.10101912733343
C	-1.43051940806760	0.00000000043188	0.95331383362459
C	-0.72390021920280	0.00000000074895	-0.28279080705818
C	0.72390021847609	0.00000000028691	-0.28279080464810
C	1.43051940733323	0.00000000039233	0.95331383571195
N	-1.26371375254839	0.00000000101508	-1.50471720398980
N	1.26371375511997	-0.00000000035628	-1.50471719996095
S	0.00000000282819	0.00000000011335	-2.57020275205031
C	2.83565508974154	-0.00000000066820	0.96184370052280
C	-2.83565509124595	-0.00000000076719	0.96184370152628
N	-4.00002091536311	-0.00000000176363	0.97051869097601
N	4.00002091369688	-0.00000000154602	0.97051868386743

BTDN^{2-}

H	1.22510014590437	0.00000002224888	3.12595333799896
C	0.67884707157025	0.00000001970418	2.18854457648176
C	-0.67884705127239	0.00000001467363	2.18854457854207
H	-1.22510013816057	0.00000001118474	3.12595333533821
C	-1.43905105565655	0.00000000842380	0.95386250806743
C	-0.72999917340414	0.00000001490337	-0.29015996002605
C	0.72999917812358	0.00000001373160	-0.29015998748997
C	1.43905108354248	0.00000000999383	0.95386249958377
N	-1.27542987408089	0.00000001086233	-1.50528523242312
N	1.27542979754114	0.00000000612470	-1.50528530249006
S	-0.00000007810110	0.00000000929893	-2.61263484914229
C	2.82809666890115	-0.00000002236239	0.97021082472304
C	-2.82809662352399	-0.00000002169687	0.97021077551646
N	-4.00321801987847	-0.00000004724038	0.99248823175046
N	4.00321806849513	-0.00000004985036	0.99248835315386

[BTDN-(H)$_2$]

H	1.23069680439803	0.00000007123837	3.12031316786929
C	0.67776779392524	-0.00000001367411	2.18991708191743
C	-0.68434461350019	-0.00000000589721	2.18744196989318
H	-1.24062638157199	0.00000008705219	3.11583714617686
C	-1.41247495167165	-0.00000011643550	0.95642818430820
C	-0.70747649880134	-0.00000007911960	-0.24910986824111
C	0.70970965913568	-0.00000000994609	-0.24654463805486
C	1.41032549142614	-0.00000007619428	0.96154105140366
N	-1.19173583645865	-0.00000006713204	-1.51602095314072
N	1.19868741472739	0.00000004859763	-1.51163858149896
S	0.00567672647935	0.00000012584739	-2.74900038572944
C	2.81924958173050	-0.00000004876931	0.95173353678731
C	-2.82137430506857	0.00000000211812	0.94172762994140
N	-3.98247025851398	0.00000010414246	0.91100134523023

N	3.98044090022343	-0.00000002491598	0.92493569190317
H	2.17584817349604	0.00000010341855	-1.76704264415188
H	-2.16789969995543	-0.00000010043059	-1.77510204961377

[BTDN-$(CO_2)_2$]$^{2-}$

C	0.98821612362499	-1.81705283339329	0.00000002508888
O	1.36675253056127	-2.97418529532054	0.00000002484138
O	1.89912578298983	-0.79224015829434	0.00000001430518
C	3.92872720894360	0.94449234555731	-0.00000001383389
O	4.44732347550928	0.80136141407292	1.12816608359411
O	4.44732365555846	0.80136198646860	-1.12816610176924
H	-2.11614845138546	3.43734225602531	-0.00000002735258
C	-1.70232232681729	2.43506964753377	-0.00000002340034
C	-0.34082170928182	2.26289292901358	-0.00000002753660
H	0.33206185674812	3.10986413601512	-0.00000003728325
C	0.18957769296034	0.94026528618381	-0.00000001433364
C	-0.69780679691092	-0.10300717435519	0.00000000396832
C	-2.11574916910895	0.00549432511920	0.00000001125525
C	-2.61861623434198	1.34395356447514	-0.00000000295440
N	-0.30158924699980	-1.42446007156414	0.00000002000846
N	-2.79637883890001	-1.14057299793112	0.00000002818021
S	-1.73375400129206	-2.45287147322841	0.00000003744171
C	-4.00352509878533	1.56769305391913	0.00000000322709
C	1.59699820253023	0.60265936020058	-0.00000001137683
N	2.55590069872911	1.42451668201949	-0.00000000068434
N	-5.15628254085056	1.73863983668019	0.00000000861452

[BTDN-$(H)_1$]$^{1-}$

H	1.22416097076328	0.02971354357952	3.12863171297299
C	0.67890489480841	0.01214774207773	2.19381343032789
C	-0.68120676339137	-0.00444927357935	2.16909690362627
H	-1.24131483073014	0.00071551397732	3.09728636448016
C	-1.41339215330995	-0.01720791928190	0.93812482418166
C	-0.70720662782722	-0.02567404808870	-0.30403914958110
C	0.73086954431378	-0.02796287492942	-0.25307585810479
C	1.42240989494488	0.00302072903168	0.95596060714428
N	-1.24474870747762	-0.01215601192960	-1.52336416694338
N	1.25598225057072	-0.10158310211721	-1.51273641618447
S	-0.03155744317713	0.03958770318039	-2.68416767193011
C	2.82735811276173	0.00950460299358	0.95143761168897
C	-2.81109016672124	0.00567858667454	0.93590452208435
N	-3.97909026731105	0.02276426412958	0.92676694565227
N	3.99191368001241	0.01003209071037	0.93280977031015
H	2.14590829207050	0.33022937877148	-1.73093074942515

[BTDN-$(CO_2)_1(H)_1$]$^{1-}$

C	-2.44941961142894	0.69866515726346	-0.35599415040908
O	-2.95216875992624	1.84500052015562	-0.32038694586376
O	-2.85901633510658	-0.36949594082991	-0.82333889952595
H	0.86438063250728	-3.46269115236753	-0.26699790103242

C	0.95785628831305	-2.38685147813732	-0.18758578457965
C	2.19141924967169	-1.79368792427945	-0.27689482330984
H	3.08281156932458	-2.38523842169202	-0.43837891677236
C	2.30025041439907	-0.39263004948636	-0.12655889761200
C	1.15840536438918	0.37143402654313	0.08630285575539
C	-0.12463602003939	-0.22366282264785	0.09689539916964
C	-0.21664133528313	-1.62513133971218	0.02990975577431
N	1.15965478258358	1.74988426669156	0.36143939617056
N	-1.12957911106352	0.69276822791724	0.27864395109643
S	-0.47270896011498	2.33533753171853	0.20045628079574
C	-1.40337191158452	-2.35096862177207	0.30901535372043
C	3.56770581008910	0.24323459744933	-0.17133831724260
N	4.60455479583236	0.75746548671688	-0.21981402624207
N	-2.26644514308831	-3.06365617897114	0.61220413179041
H	1.52826708662570	1.94220857894008	1.29095465481683

$[BTDN\text{-}(CO_2)_3]^{2-}$

H	1.08991169992700	-0.42781485169147	3.29132306583179
C	0.59303459033389	-0.29245901726784	2.33872849988127
C	-0.77636267082087	-0.21013515857924	2.28363809126534
H	-1.37352522392979	-0.28197398945340	3.18321498973318
C	-1.40825140297142	-0.02072712736119	1.03977714879019
C	-0.61263510015741	0.06417752739573	-0.08166701325604
C	0.79065429014113	-0.02932956190124	-0.07013502802161
C	1.41331754787423	-0.19837019941527	1.17755849360129
N	-1.12913604634743	0.26330219599024	-1.34153248062687
N	1.28095098094600	0.09403700344960	-1.34115846232283
S	0.12602184711284	0.26210707024337	-2.63779527463426
C	2.81179656359578	-0.25000754032169	1.40281187554383
C	-2.84209590857197	0.10019738216796	0.84691482957819
N	-3.70584851208822	0.04876875628213	1.76252338330022
N	3.91711649511006	-0.28618026732693	1.75141008639217
C	-2.44814555246642	0.36368192464325	-1.59048644991247
O	-2.92980703811874	0.51068038869138	-2.69709485724901
O	-3.27088094063087	0.29256897623868	-0.49285184356733
C	2.52846017736870	-0.06217651022722	-2.00880842708114
O	3.56778894589505	-0.28254899743727	-1.39418810944732
O	2.29622801569607	0.07045885389392	-3.25795920535694
C	-5.12101083743295	0.18614514847901	1.44353956509890
O	-5.72376315088252	-0.88637024825723	1.23546442284848
O	-5.56471474818210	1.35150233516475	1.49085172441097

$[BTDN\text{-}(CO_2)_2]^{3-}$

C	0.98662944172182	-1.79179129134095	-0.00003278960960
O	1.38344011374571	-2.95237424433627	-0.00005589320376
O	1.90247858844989	-0.77519250051734	-0.00002093755124
C	3.92660890903823	0.91454087289228	0.00002436352868
O	4.46948903564936	0.75018345156191	1.11879611766721
O	4.46954680330571	0.75018221130051	-1.11872490346023
H	-2.12164170303446	3.44000744947352	0.00001726782729
C	-1.71190834219063	2.43630627827254	0.00000846496185

C	-0.35473554491353	2.27208200260984	-0.00002622398052
H	0.29683954898685	3.13609733556556	-0.00003050295093
C	0.21476380232162	0.95584834368440	-0.00002723361872
C	-0.71978811082848	-0.09067695169659	0.00001545488906
C	-2.10451677924680	0.00430104371676	0.00002729961169
C	-2.64899725201344	1.33981974078907	0.00000003916362
N	-0.28925777805369	-1.41842550823719	0.00000374910199
N	-2.80541430615550	-1.15073436468593	0.00003054303911
S	-1.70959808993686	-2.44507037372685	0.00003522487164
C	-4.01696254162976	1.55083609926090	-0.00000140029899
C	1.58633253685966	0.62914932454707	-0.00002707924906
N	2.59229561596347	1.42822845271236	-0.00000996831774
N	-5.17659113455812	1.72789944735151	-0.00000159242136

$[BTDN-(CO_2)_4]^{2-}$

H	1.28545750646842	0.00685056375000	3.51558321712369
C	0.72360539538257	0.00672871595190	2.59015487904220
C	-0.64277518246106	0.02534906450615	2.60571449609998
H	-1.18308181552131	0.06740112967667	3.54292504103167
C	-1.38425629207431	-0.02050967640226	1.39278573914849
C	-0.69494589594604	-0.10547043755886	0.17611682619195
C	0.71986795232436	0.02801071904136	0.15663755337451
C	1.43723457746552	-0.00271350234056	1.35980540354656
N	-1.21907089617788	-0.25660838610468	-1.08053711967608
N	1.21324848921990	0.12112716348700	-1.11759555938341
S	-0.01847908940683	-0.08928035997828	-2.34441728732997
C	2.84125259086318	-0.16728768782761	1.41674248242108
C	-2.78693573678393	0.14519270044144	1.47292550241214
N	-3.91044095934801	0.37733378158040	1.65023921515113
N	3.96730489426760	-0.39598493302537	1.58200645451313
C	-2.34636688819486	-1.07903889533784	-1.50223830969069
O	-2.45117226552320	-1.11179505594254	-2.74886565403232
O	-3.01707361728410	-1.61363274966323	-0.60799543515352
C	2.33754316961613	0.91031891807611	-1.60243622678509
O	3.04163378705977	1.46795897204947	-0.74884441478622
O	2.40580793926749	0.89498784789102	-2.85217980150433
C	-5.38398628740413	-0.96797520595904	-2.16094801539096
O	-5.23252055932774	0.14942026520956	-1.87749527220877
O	-5.59091579955368	-2.07278858308393	-2.45812563036817
C	5.35446853958780	0.82358545177946	-2.37116611255277
O	5.23923725250190	-0.28768837136055	-2.04912412207753
O	5.52717696778244	1.92252296754423	-2.70939794921670

References

[1] M. Más-Montoya, D. Curiel, C. Ramírez De Arellano, A. Tárraga, P. Molina, *Eur J Org Chem* **2016**, *2016*, 3878–3883.
[2] A. Salamé, M. Hon Cheah, J. Bonin, M. Robert, E. Anxolabéhère-Mallart, *Angew Chem Int Ed* **2024**, *63*, e202412417.
[3] C. Yang, Y. Fu, Y. Huang, J. Yi, Q. Guo, L. Liu, *Angewandte Chemie* **2009**, *121*, 7534–7537.

[4] M. Axelsson, C. F. N. Marchiori, P. Huang, C. M. Araujo, H. Tian, *J. Am. Chem. Soc.* **2021**, *143*, 21229–21233.
[5] C. Dainty, D. W. Bruce, D. J. Cole-Hamilton, P. Camilleri, *J. Chem. Soc., Chem. Commun.* **1984**, 1324.
[6] P. Camilleri, A. Dearing, D. J. Cole-Hamilton, P. O'Neill, *J. CHEM. SOC. PERKIN TRANS.* **1986**.
[7] J. N. Robinson, D. J. Cole-Hamilton, P. Camilleri, *J. Chem. Soc., Faraday Trans. 1* **1989**, *85*, 3385.
[8] J. N. Robinson, D. J. Cole-Hamilton, M. K. Whittlesey, P. Camilleri, *Faraday Trans.* **1990**, *86*, 2897.
